# Disclosing the Potential of Fluorinated Ionic Liquids as Interferon-Alpha 2b Delivery Systems

**DOI:** 10.3390/nano12111851

**Published:** 2022-05-28

**Authors:** Margarida L. Ferreira, Nicole S. M. Vieira, Ana L. S. Oliveira, João M. M. Araújo, Ana B. Pereiro

**Affiliations:** LAQV, REQUIMTE, Department of Chemistry, NOVA School of Science and Technology, NOVA University Lisbon, 2829-516 Caparica, Portugal; mal.ferreira@campus.fct.unl.pt (M.L.F.); ns.vieira@campus.fct.unl.pt (N.S.M.V.); als.oliveira@campus.fct.unl.pt (A.L.S.O.)

**Keywords:** therapeutic proteins, ionic liquids, aggregation behaviour, interactions, delivery systems

## Abstract

Interferon-alpha 2b (IFN-α 2b) is a therapeutic protein used for the treatment of cancer, viral infections, and auto-immune diseases. Its application is hindered by a low bioavailability and instability in the bloodstream, and the search for new strategies for a target delivery and stabilization of IFN-α 2b to improve its therapeutic efficacy is crucial. Fluorinated ionic liquids (FILs) are promising biomaterials that: (i) can form self-assembled structures; (ii) have complete miscibility in water; and (iii) can be designed to have reduced toxicity. The influence of IFN-α 2b in the aggregation behaviour of FILs and the interactions between them were investigated through conductivity and surface tension measurements, and using electron microscopic and spectroscopy techniques to study FILs feasibility as an interferon-alpha 2b delivery system. The results show that the presence of IFN-α 2b influences the aggregation behaviour of FILs and that strong interaction between the two compounds occurs. The protein might not be fully encapsulated by FILs. However, the FIL can be tailored in the future to carry IFN-α 2b by the formation of a conjugate, which prevents the aggregation of this protein. This work constitutes a first step toward the design and development of FIL-based IFN-α 2b delivery systems.

## 1. Introduction

Over recent decades, the pharmaceutical industry has experienced remarkable exponential growth in the approval of new biological therapeutical entities, while the development of traditional small drugs has reached a stagnated phase [1,2,3,4]. The prospect of having biological products for therapeutic ends has revolutionized drug discovery and development, opening unprecedented avenues of research for biopharmaceuticals [1,2,3,4]. These compounds have contributed to the diagnosis and treatment of several diseases where conventional therapies have constantly failed, such as numerous cancers, immune and autoimmune infections and metabolic diseases, Alzheimer’s, HIV, and Parkinson’s, along with others [3,4]. Among biopharmaceuticals, therapeutic proteins arise with a vast availability, specific action mode, and impressive characteristics such as high specificity, safety, tolerability, efficiency, and a low prospect of unspecific and drug-drug interaction [5,6]. These properties make them unique compounds to formulate a huge number of therapeutic protein-based treatments with regulatory and special targeting activity, vaccines, and diagnostics [6]. However, their application in the pharmaceutical industry is limited by their intrinsic properties. Therapeutic proteins are very sensitive to: (i) alterations in the conformation of the structure; (ii) interactions with the excipients on the formulation; (iii) impurities from the process of manufacture; (iv) storage conditions that can cause degradation or aggregation; (v) the dosage and time needed for therapeutic effect; (vi) the route of administration; and (vii) the genetic characteristics of the patient [6,7]. All these factors hamper the in vivo delivery of therapeutic proteins and make them chemically and physically unstable compounds with a short half-life in the bloodstream and consequently poor bioavailability. This handicap is commonly compensated for by the administration of regular dosages or higher amounts of the drug to obtain a therapeutic effect [8,9,10]. However, prolonged exposure to the biopharmaceutical combined with the increased dosages and sensitivity problems leads to significant adverse side effects, toxicity, and immunogenicity [8,9,10].

To overcome these complications, the development of efficient strategies to extend the half-life of therapeutic proteins through the administration routes has been in the research spotlight [10]. From those strategies, significant advances have been attained using drug delivery systems (DDSs) that aid the control of stability and release of the therapeutic proteins, protecting and maintaining their activity, improving their bioavailability, and guaranteeing their safety [10,11,12,13,14]. Amongst the different types of DDSs that have been developed, nanocarriers have found a place as promising tools to circumvent those challenges. They have been demonstrated to be non-invasive, safe, and targeted delivery methods [12,13,14,15]. However, there are still concerns from a biological and technological point of view that limit the usage of these systems, such as low stability, uncontrolled release of proteins, low encapsulation efficiency, expensive costs associated with the formulation and production of these systems, and difficulties in the scaling up of the process [12,13,14,15]. Thus, even though some DDSs are already available in the market [15], there are still no fully effective and profitable nanocarriers.

Interferon-alpha 2b (IFN-α 2b) is one of the most relevant therapeutic proteins under research in the past decades. This protein belongs to the class of type I interferons, which are cytokines with a key role in the innate immune response [16,17]. Therefore, IFN-α 2b has a highly immunomodulatory response, being used for the treatment of several cancers, viral infections, and auto-immune diseases [16,17,18,19,20]. It is already approved to be used in the treatment of leukaemia, multiple myeloma, carcinoma, and hepatitis B and C, among others [20]. More recently, strong evidence has suggested the use of IFN-α 2b in the mitigation of severe clinical problems caused by COVID-19, such as pneumonia [21,22]. This therapeutic protein is usually administrated subcutaneously and intramuscularly to avoid proteolysis [16,23]. Nevertheless, alternative routes or new mechanisms must be considered to improve the bioavailability of this protein. IFN-α 2b easily degrades and has a short half-life in the organism (2 to 3 h), being quickly eliminated from the bloodstream [18,23]. It must be then systematically administrated to have a clinical effect, or the dosage must be elevated, which results in significant toxicity, triggering severe adverse reactions and limiting its usage in elderly and debilitated patients [23,24]. Therefore, the development of DDSs to provide more efficient and safer IFN-α 2b formulations is of special attention for clinical applications. Several DDSs for transporting IFN-α have been developed, such as pegylated forms, liposomes, polymeric micelles, microencapsulation, and nanoencapsulation, along with others [23]. Despite the efforts to search liable DDSs for interferons, none of them have been approved for clinical usage in humans [23], and the research for safer IFN-α 2b formulations must continue.

Ionic liquids (ILs) are considered alternative biomaterials which have been in the spotlight for several applications, having attained in recent years an important place in biological, biotechnological, medical, and pharmaceutical fields [25,26]. ILs’ structure can be manipulated to obtain compounds with outstanding properties, increased biocompatibility and biodegradability, which have drawn the attention of several researchers to their biological activity [25,26]. The use of ILs as DDSs has been under investigation due to their capacity to stabilize and enhance the solubilization of macromolecules such as proteins [27,28], especially the surface-active ionic liquids (SAILs) [29,30]. Fluorinated ionic liquids (FILs) are a family of SAILs and have shown promising results in the biopharmaceutical field [31,32]. These compounds are characterized by the introduction of perfluoroalkyl chains with equal or more than four carbon atoms in the cation and/or anion. This structural feature can promote the formation of three nano-segregated domains (one polar and two non-polar: hydrogenous and fluorous), which grant improved properties to FILs [31,32,33,34,35,36]. They are mainly characterized by impressive surfactant power, chemical and biological inertness, low surface tension, negligible vapour pressure, tuneability, and high thermal stability, along with other excellent properties that are common in the conventional ILs and perfluoroalkyl compounds, such as high solubility of gases [31,32,33,34,35,36,37]. FILs constituted by perfluoroalkyl anions with only four carbon atoms have shown enhanced self-aggregation behaviour that aids the formation of self-assembled structures in aqueous solutions [31,32,38], which combined with the low surface tension [31] make FILs highly surface-active compounds. This superior surfactant behaviour allows those FILs to be completely miscible in aqueous solutions [38]. Moreover, FILs can be customized to have negligible cytotoxicity in four different human cell lines [31,32,39,40], high acceptance of red blood lysis [32], and insignificant ecotoxicity to several aquatic species and bacteria [31,32]. These properties have been key to considering the use of FILs in the biological field, especially in the formulation of DDSs for therapeutic proteins. Some works have taken an important step toward this purpose. Alves et al. [41] have studied the development of protein drug delivery systems using biocompatible FILs and lysozyme. They have proven for the first time that FILs can encapsulate this protein without affecting the stability and biological activity of this macromolecule [41]. Vieira et al. [42] have demonstrated that after the encapsulation of lysozyme by FILs, the system is stable and the protein can be later released with the application of external stimuli, maintaining the properties of the protein intact. Ferreira et al. [40] have also successfully encapsulated lysozyme with hydroxyl functionalized FILs (more biocompatible compounds), keeping the activity of this protein. Alves et al. [43] also studied the impact of FILs on the stability, structure, and interactions of bovine serum albumin (BSA). The results showed that the self-assembled structures of FILs have strong interactions with BSA, and they can be considered a promising potential novel biomaterial for drug delivery systems using therapeutic proteins [43]. Recently, the study of the interactions between FILs and human phenylalanine hydroxylase protein has also demonstrated the encapsulation of the protein by the FIL aggregates [44]. Therefore, the encapsulation and stabilization of therapeutic proteins using FILs have demonstrated to be a promising strategy to overcome the handicaps in the delivery of therapeutic proteins in the human body, allowing the improvement of their bioavailability, safety, and efficacy by preserving the structure, stability, and biological activity of several different biomolecules.

In this work, a first step was taken in the study of FILs as a tool to stabilize and deliver IFN-α 2b. To obtain insights into the mechanism of FILs’ solubilization of this specific therapeutic protein, the protein-FIL interactions were under investigation. From the literature, only two works have covered the study of ILs with IFN-α 2b. The first work studied protein purification by aqueous two-phase systems using ILs as adjuvants [45]. Very recent work has also screened several FILs for IFN-α 2b to understand which structural features can interact with the protein, obtaining promising results in the ones based on the cholinium cation [46]. Therefore, two FILs based on a perfluorobutanesulfonate anion ([C_4_F_9_SO_3_]^−^) conjugated with cholinium and imidazolium cations were selected in this work to obtain insights into protein-FIL interactions. Then, the aggregation behaviour of the FILs was investigated under the presence of IFN-α 2b by: (i) the determination of the critical aggregation concentrations (CACs); (ii) assessment of the surface properties; and (iii) insights into the morphology of the FILs’ self-assembled aggregates using microscopic and spectroscopy methods. Moreover, the interactions of FILs with the IFN-α 2b were studied using different methods such as ultraviolet-visible, fluorescence, and circular dichroism spectroscopies. Finally, the binding of the FILs to IFN-α 2b was also accessed using microscale thermophoresis. The results obtained in this work open new paths to the investigation of DDSs based on FILs for the delivery and stabilization of valuable therapeutic proteins such as IFN-α 2b.

## 2. Materials and Methods

### 2.1. Materials

Interferon-alpha 2b (IFN-α 2b), human recombinant, SRP4595, expressed in *E. coli* (≥98% mass fraction purity by SDS-PAGE and HPLC) was purchased from Sigma-Aldrich (Saint Louis, MO, USA). Different concentrations of the protein were used depending on the sensitivity of each method. The concentrations used are detailed in each method description. Sodium chloride (NaCl, ≥99.5% mass fraction purity) from Merck (Darmstadt, Germany), potassium dihydrogen phosphate (KH_2_PO_4_, ≥99.0% mass fraction purity), sodium dihydrogen phosphate anhydrous (NaH_2_PO_4_, ≥99.0% mass fraction purity), hydrochloric acid (HCl) at 0.1 M from Fluka (Charlotte, NC, USA) and Milli-Q water from in-house laboratory facilities were used for the preparation of buffers. In addition, 1-Ethyl-3-methylimidazolium perfluorobutanesulfonate [C_2_C_1_Im][C_4_F_9_SO_3_] (≥97% mass fraction purity), and (2-hydroxyethyl)trimethylammonium perfluorobutanesulfonate [N_1112(OH)_][C_4_F_9_SO_3_] (>97% mass fraction purity) were provided by IoLiTec GmbH (Heilbronn, Germany). Both FILs were verified by ^1^H and ^19^F NMR spectroscopy (NMR spectrometer, Bruker 400 MHz) and dried under vacuum (4 Pa) and vigorous stirring for at least 48 h at 323.15 K before usage to ensure the absence of volatile substances and a water content lower than 100 ppm, confirmed by the Karl Fisher coulometric titration method (Metrohm 831 KF Coulometer). The nomenclatures and structures of FILs are represented in Table 1.

### 2.2. Conductivity Titration Experiments

The critical aggregation concentrations of [C_2_C_1_Im][C_4_F_9_SO_3_] were determined by an ionic conductivity titration method in the range of 0 to ~130 mmol kg^−1^ in the presence of different biological simulated fluids and IFN-α 2b. For this purpose, a CDM210 conductometer (Radiometer Analytical, Lyon, France) and a CDC749 electrode (Radiometer Analytical, Lyon, France) were utilized. The electrode was calibrated at 25 °C using two standard solutions of 0.1 and 0.01 D KCl (Radiometer Analytical). The standard solutions and samples were measured inside of a glass cell in a thermostated bath at 25 °C with constant magnetic stirring. The temperature was maintained and registered using a platinum resistance thermometer attached to a Keithley 199 system DMM/scanner (uncertainty of ±0.1 °C) from Keithley Instruments (Solon, OH, USA). Six samples of ~130 mmol kg^−1^ [C_2_C_1_Im][C_4_F_9_SO_3_] were prepared and used to start each system in: (i) 150 mM of NaCl (pH = 7.3) to simulate the ionic strength and pH of the bloodstream; (ii) 25 mM of KH_2_PO_4_ (pH = 6.8) to mimic the intestinal fluid; (iii) 100 mM of HCl (pH = 1.2) to replicate the gastric fluid; (iv) 150 mM and (v) 5 mM (the two concentrations used in this work, depending on the used method) of NaH_2_PO_4_ (pH = 7.4) as the buffer of IFN-α 2b; and finally (vi) 5 mM of NaH_2_PO_4_ (pH = 7.4) with 10 μg/mL of IFN-α 2b (the mixture of protein with FIL in the buffer of the IFN-α 2b). Each sample was then placed on the cell, stirred, and had its ionic conductivity measured. Afterwards, different amounts of buffer and/or solutions of buffer with protein (10 μg/mL of IFN-α 2b) were titrated to the initial solution, stirred, and the ionic conductivity was measured. This experimental procedure was carried out until the ionic conductivity reached a plateau. After that, a solution of buffer and/or buffer with protein (10 μg/mL of IFN-α 2b) was placed inside the cell and different amounts of a solution with a known concentration of FIL and/or FIL with protein (10 μg/mL of IFN-α 2b) were added to have the complete conductivity profile of each system. The ionic conductivity was measured at least three times before adding more solution and the uncertainty of each measurement was estimated to be less than 1%.

### 2.3. Density Measurements

The density of [C_2_C_1_Im][C_4_F_9_SO_3_] + water in a range of 0 to ~730 mM was assessed using an automated SVM 3000 Anton Paar rotational Stabinger viscometer-densimeter. The measurements were performed at atmospheric pressure and 25 °C. This apparatus utilizes Peltier elements guaranteeing an uncertainty of ±0.02 °C in the temperature. The density has an uncertainty of 2 × 10^−4^ g cm^−3^ between triplicates.

### 2.4. Tensiometry and Contact Angle Goniometry

Several solutions of [C_2_C_1_Im][C_4_F_9_SO_3_] in the range of 0 to ~240 mM were prepared in 5 mM of NaH_2_PO_4_ (pH = 7.4, buffer of the IFN-α 2b) and in 5 μg/mL of IFN-α 2b with 5 mM of NaH_2_PO_4_ (pH = 7.4). The surface tension of each solution was determined at 25 °C by the Du Noüy ring method in a KSV’s Sigma 702 Tensiometer (Biolin Scientific, Gothenburg, Sweden). The force tensiometer is equipped with a platinum Du Noüy ring to surface tension measurement and a thermostatic vessel connected to a bath circulator (RW-0535G, Lab. Companion, Daejeon, Republic of Korea) in order to keep the temperature of the solution constant. Next, 15 mL of each sample was placed in a glass container and left to equilibrate in the thermostatic vessel. After temperature equilibration, the surface tension was measured. The equipment is a standalone-controlled instrument, and the calibration was conducted with a calibration weight with a known mass before measuring the samples. Each sample was measured five times, and the reported surface tension was the average value with an uncertainty of ±0.22 mN/m. Each solution was then assessed by a KSV’s contact angle goniometer CAM100 (Biolin Scientific, Gothenburg, Sweden). A drop of each solution was placed on a Teflon surface with a Hamilton syringe. This equipment is a PC-controlled instrument, and the tilt was set to 0 degrees at the base of the drop and was recorded for 10 frames with an interval of 1 s. The contact angle was calculated for the left and right sides of the drop and the mean value was the resulting contact angle. At least five different drops of each solution were recorded, and the reported contact angle was the resulting average with an uncertainty of ±5%.

### 2.5. Scanning Transmission Electron Microscopy with Energy-Dispersive X-ray Spectroscopy

The scanning transmission electron microscopy (STEM) with energy-dispersive X-ray spectroscopy (EDS) microanalysis was performed in a Dual-Beam FEI Helios Nanolab microscope, at a working voltage of 15 kv with a dark field detector. Three solutions were prepared to measure the blank of the protein, the blank of the FIL and the mixture of protein with FIL in the buffer of the IFN-α 2b: (i) IFN-α 2b at 0.0001 μg/mL in 150 mM of NaH_2_PO_4_ (pH = 7.4); (ii) [C_2_C_1_Im][C_4_F_9_SO_3_] at 29.2 mM in 150 mM of NaH_2_PO_4_ (pH = 7.4) and (iii) IFN-α 2b at 0.0001 μg/mL with [C_2_C_1_Im][C_4_F_9_SO_3_] at 29.2 mM in 150 mM of NaH_2_PO_4_ (pH = 7.4). The samples were placed on a 200-mesh copper grid (3 mm diameter). The excess sample was removed using filter paper and samples were dried before measurement.

### 2.6. Dynamic Light Scattering

The dynamic light scattering (DLS) of [C_2_C_1_Im][C_4_F_9_SO_3_] and [N_1112(OH)_][C_4_F_9_SO_3_] in the presence of IFN-α 2b was recorded using a Zetasiser Nano Series ZEN3600 (Malvern, UK). The apparatus is equipped with a 633 nm laser and with a non-invasive backscattering technique (173°) used for detection. Stock solutions of FILs and IFN-α 2b were prepared in the buffer of the IFN-α 2b, 150 mM of NaH_2_PO_4_ (pH = 7.4), and filtered through filters with a pore diameter of 0.22 μm. Solutions of FILs + IFN-α 2b were prepared and adjusted with filtered buffer to final concentrations of 29.3 and 243.8 mM for [C_2_C_1_Im][C_4_F_9_SO_3_], 29.8 and 248.0 mM for [N_1112(OH)_][C_4_F_9_SO_3_] and 50 μg/mL of IFN-α 2b. These concentrations were chosen to comprise two different aggregates of each FIL, above the 1st CAC and the 3rd CAC. The solutions were left to equilibrate for 24 h at 4 °C to promote the interaction of the protein with FILs. Therefore, 20 μL was transferred into a quartz cell of 10 mm pathlength and measured at 25 °C.

### 2.7. Ultraviolet-Visible Spectroscopy

Absorbance measurements were performed with a double beam ultraviolet-visible (UV–Vis) spectrophotometer (UV-6300PC) from VWR (Radnor, PA, USA). Solutions of [C_2_C_1_Im][C_4_F_9_SO_3_] (7.3, 26.6, 59.7, 106.3 and 425.1 mM) and [N_1112(OH)_][C_4_F_9_SO_3_] (8.2, 25.8, 110.8, 192.2 and 384.4 mM) were prepared in the buffer of the protein, 5 mM of NaH_2_PO_4_ (pH = 7.4), with 20 μg/mL of IFN-α 2b and without the protein (blanks). These concentrations were selected to comprise all the aggregation stages of the FIL: below the 1st CAC, between the 1st and 2nd CACs, 2nd and 3rd CACs, 3rd and 4th CACs, and above the 4th CAC. The solutions were prepared, left to equilibrate for 30 min at room temperature, and measured (time 0 h). After that, they were incubated at 4 °C for 24 h to allow the FIL to interact with the protein and measured once again (time 24 h). Then, 400 μL of each solution (sample and respective blank) was transferred to a matched pair of quartz cuvettes (10 mm path length) and assessed in a wavelength range between 190 and 400 nm. For protein samples, a buffer as blank was used, while for the samples of FIL + protein, the solutions of FIL were used as blank. Each solution was measured at least three times with an error of ±5%.

### 2.8. Fluorescence Spectroscopy

The intrinsic fluorescence of IFN-α 2b was determined using a spectrofluorometer Spex Horiba Jobyin-Yvon (Kyoto, Japan) making use of the protein tryptophan residues as intrinsic fluorophores. Several solutions of [C_2_C_1_Im][C_4_F_9_SO_3_] (2, 3.7, 7.3, 11, 14.6, 26.6, 59.7, 93.6, 221.6, 443.2, 886.4 mM) and [N_1112(OH)_][C_4_F_9_SO_3_] (2, 4, 8.2, 10.7, 16.1, 25.8, 51.3, 77.1, 110.8, 151.5, 192.2, 288.4, 384.4, 576.6, 768.7 mM) were set in the buffer of the protein, 5 mM of NaH_2_PO_4_ (pH = 7.4), with and without (blanks) the presence of IFN-α 2b (20 μg/mL). The concentrations were chosen to cover the range where the different aggregates of the FILs are formed. Samples, equilibrated for 30 min at room temperature, and 400 μL of each solution were transferred to a quartz cuvette of 10 mm pathlength and excited at 280 nm, collecting the fluorescence intensity in a wavelength range of 300 to 450 nm. The width of the excitation and emission slit was set to 5 nm for both cases. The spectra of FIL solutions (blanks) were discounted to the spectra of FIL + protein samples to avoid any possible effect of FIL concentration increment in the fluorescence intensity.

### 2.9. Circular Dichroism Spectroscopy

The circular dichroism (CD) spectra were acquired by Chirascan spectropolarimeter (Applied Photophysics, UK) for solutions of IFN-α 2b (100 μg/mL) with [N_1112(OH)_][C_4_F_9_SO_3_] at 248 mM in the buffer of the protein (150 mM of NaH_2_PO_4_, pH = 7.4). The concentration of FIL was chosen to cover the maximum of FIL that can be used for the studied application. Stock samples of FIL and protein were prepared and filtered through a 0.2 mm filter. Afterwards, they were used to prepare the solutions of protein and protein with FIL at the defined concentrations. Samples were left to equilibrate for 30 min at room temperature and around 230 μL of each sample was moved to a cuvette with 1 mm of path length. CD spectra were expressed in millidegrees and obtained in a range of 200 nm to 260 nm by three consecutive readings at constant temperature (25 °C). Spectral deconvolution was executed with K2D3 [47] to allow for the estimation of the secondary structure of the protein with and without the presence of FILs.

### 2.10. Microscale Thermophoresis

IFN-α 2b binding affinity with [C_2_C_1_Im][C_4_F_9_SO_3_] and [N_1112(OH)_][C_4_F_9_SO_3_] were determined by microscale thermophoresis (MST) using a Monolith NT.115 (BLUE/RED, NanoTemper Technologies, Munich, Germany). The IFN-α 2b was labelled fluorescently by a protein labelling kit, Alexa Fluor^TM^ 555 (A20174, Invitrogen, Thermofisher Scientific Inc., Waltham, MA, USA), according to instructions from the manufacturer. Several dilution series of 16 samples of 20 μL were prepared in triplicate for each studied system. All the solutions were prepared in the buffer of the protein, 5 mM of NaH_2_PO_4_ (pH = 7.4), and PCR tubes. In the first tube (1 out 16) 20 μL of FIL stock solution was added at twofold the maximum concentration defined for each assay. In the remaining 15 tubes, 10 μL of buffer was added, followed by the series dilution of the FIL. Therefore, 10 μL of labelled IFN-α 2b stock solution was added to each tube yielding a final protein concentration of 2.7 μM. For [C_2_C_1_Im][C_4_F_9_SO_3_], five maximum concentrations were selected (14, 30, 80, 160, 220 mM), while for [N_1112(OH)_][C_4_F_9_SO_3_], four maximum concentrations were chosen (15, 35, 70, 375 mM). The selected concentrations cover the range where the distinct CACs are formed in each FIL. The excitation power was determined by a pre-test and set to 20% whereas the MST power was set to medium (standard). The assays were executed in standard capillaries and triplicates. The results were processed using the MO.Affinity Analysis v2.3 software (NanoTemper Technologies, Munich, Germany).

## 3. Results and Discussion

### 3.1. Influence of Interferon-Alpha 2b in the Aggregation Behaviour of Fluorinated Ionic Liquids

FILs have great potential to be used as drug delivery systems of IFN-α 2b due to their rich aggregation behaviour in aqueous solutions, which grants improved mechanisms of solvation and complete water miscibility. Therefore, the behaviour of the self-assembled structures of FILs in the presence of IFN-α 2b was disclosed in this work by using different strategies. To begin, the critical aggregation concentrations of FILs’ aqueous solutions were determined in the presence of different media and IFN-α 2b. In addition, the surface properties were accessed by the measurement of the surface tension and contact angles of the FILs’ aqueous solutions in the presence of IFN-α 2b. Finally, the self-assembled structures of FILs with IFN-α 2b were characterized through scanning transmission electron microscopy and dynamic light scattering.

To study the influence of IFN-α 2b on the aggregates of FILs, two FILs were selected: [C_2_C_1_Im][C_4_F_9_SO_3_] and [N_1112(OH_][C_4_F_9_SO_3_]. These FILs have outstanding surface-active properties, full miscibility in water, and negligible toxicity [31,32,38]. Both FILs can form different aggregates that were identified by the determination of distinct CACs in water [38]. These self-assembled structures were characterized through different techniques, where different sizes and shapes were determined. The 1st CAC represents the transition from monomers to spherical micelles, the 2nd CAC from spheric to globular micelles, and the 3rd CAC from globular to cylindrical or lamellar micelles [38]. In the case of [C_2_C_1_Im][C_4_F_9_SO_3_], an extra transition above the 3rd CAC was observed and was identified as a 4th CAC, but no description of the structure shape was made due to experimental limitations [38]. Hence, these FILs have improved aggregation behaviour when compared with the traditional surfactants, which commonly have only one critical micellar concentration (CMC, which in our case we identified as the 1st CAC) [30]. Taking advantage of these different aggregates, in this work, we evaluate the influence of IFN-α 2b on their behaviour and their proficiency as a drug delivery system for this protein.

#### 3.1.1. Critical Aggregation Concentrations

To understand the impact of different simulated biological fluids, protein medium, and IFN-α 2b on the aggregation behaviour of FILs, the [C_2_C_1_Im][C_4_F_9_SO_3_] was selected. This FIL has aggregates very well characterized in pure water [38] and with other biomolecules, such as BSA and lysozyme [40,41,42,43]. One way to acquire this information is through the careful analysis of the FIL conductivity profile, which is dependent on the FIL concentration. As mentioned before, [C_2_C_1_Im][C_4_F_9_SO_3_] has four different CACs; however, only three can be determined using conductometric titration [38]. In this work, the ionic conductivity of [C_2_C_1_Im][C_4_F_9_SO_3_] was measured in a concentration range between 0 and 130 mmol kg^−1^ with different simulated biological fluids at 25 °C to study the influence of the distinct CACs and, consequently, on the different FIL aggregates. For that purpose, six different conditions were selected: (i) 150 mM of NaCl (pH = 7.3) to mimic the pH and ionic strength of blood; (ii) 25 mM of KH_2_PO_4_ (pH = 6.8) to replicate the intestine environment; (iii) 100 mM of HCl (pH = 1.2) to simulate the gastric fluid acidity; (iv) 150 mM and (v) 5 mM of NaH_2_PO_4_ (pH = 7.4), which are both the media/buffer used for IFN-α 2b and the two concentrations used in this work (depending on the method used); and (vi) 10 μg/mL of IFN-α 2b in 5 mM of NaH_2_PO_4_ (pH = 7.4) to understand the influence of the protein in the aggregates of the FIL. The study of the FIL aggregation behaviour in these fluids allows for finding how the different aggregates can be influenced by the protein and the impact of the possible administration routes mostly used for biopharmaceuticals.

The conductivity profiles determined for all the systems can be found in Figure S1 of the Supplementary Materials (SM), as well as the one in water [38] for comparison proposes. As expected, the value of conductivity is dependent on the concentration of salt and acid, which increment in the following order: water < IFN-α 2b in 5 mM of NaH_2_PO_4_ (pH = 7.4) ≈ 5 mM of NaH_2_PO_4_ (pH = 7.4) < 25 mM of KH_2_PO_4_ (pH = 6.8) < 150 mM of NaH_2_PO_4_ (pH = 7.4) < 150 mM of NaCl (pH = 7.3) < 100 mM of HCl (pH = 1.2). The higher concentration of salts in the buffers raises the conductivity value, as well as the acidity of the solution. This behaviour is related to the high number of ions and higher mobility of these ions in the solution, which increase the conductivity values. This profile is typical of surfactants and the formation of aggregates hampers the mobility of the ions that were solvated and/or free in the solution after the process of aggregation begins. The same behaviour is not found for the acidic solution, which after a very sharp transition reaches a plateau, and the conductivity is constant along with the increment in FIL concentration. Finally, the systems with and without protein in the same buffer (5 mM of NaH_2_PO_4_) have similar behaviour.

**Figure 1:**
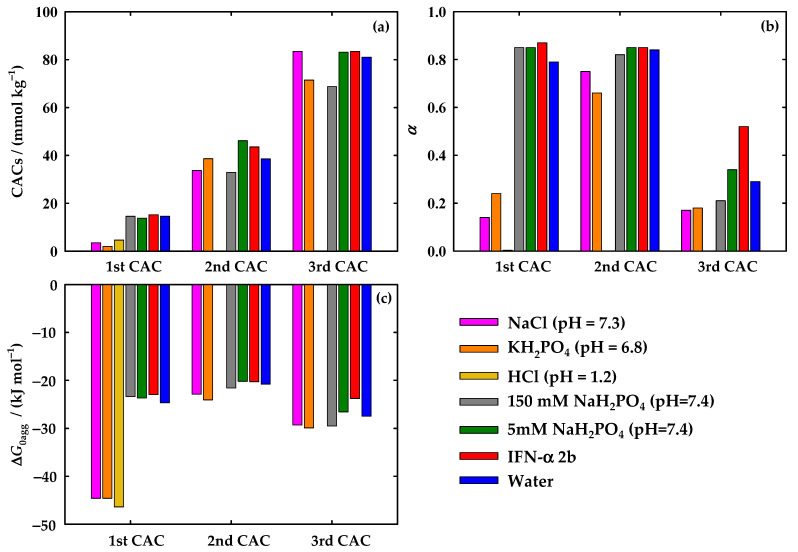
(**a**) Critical aggregation concentrations (CACs), (**b**) ionization degree (*α*), and (**c**) Gibbs free energy of aggregation ($\Delta G_{\mathrm{agg}}^{0}$) of [C_2_C_1_Im][C_4_F_9_SO_3_] at 25 °C in different conditions: 150 mM NaCl at pH 7.3 (pink); 25 mM KH_2_PO_4_ at pH 6.8 (orange); 100 mM HCl at pH 1.2 (yellow); 150 mM of NaH_2_PO_4_ at pH 7.4 (grey); 5 mM of NaH_2_PO_4_ pH 7.4 (green); 10 μg/mL mM of IFN-α 2b in 5 mM of NaH_2_PO_4_ at pH 7.4 (red); and water (blue) [38] for comparison purposes.

A thorough analysis of the conductivity profile was advanced by applying the Phillips definition [48], which studies the change of the slopes of the conductivity curve along with the variation in FIL concentration. Three different CACs were determined for almost all the studied systems, as previously obtained in water [38], except for the one of [C_2_C_1_Im][C_4_F_9_SO_3_] in HCl where only one transition was found. The determined CAC values are shown in Table S1 of Supplementary Materials and Figure 1a, as well as the ones of water [38], which are used as a reference for the [C_2_C_1_Im][C_4_F_9_SO_3_] surfactant behaviour in this discussion. Analysing each transition, Figure 1a shows that the 1st CAC, for the case of the three systems simulating the biological fluids (25 mM KH_2_PO_4_ (pH = 6.8) < 150 mM NaCl (pH =7.3) < 100 mM HCl (pH =1.2)), occurs in a lower concentration of FIL when compared with the reference (water). This behaviour was not found in the case of the systems with the buffer of IFN-α 2b. These CAC values are very close to the one of water and the concentration of NaH_2_PO_4_ does not significantly affect the 1st CAC. Furthermore, the presence of IFN-α 2b did not impact the value of this 1st CAC, which is very similar to the reference. Therefore, we can conclude that the simulated biological fluids favour the aggregation of [C_2_C_1_Im][C_4_F_9_SO_3_].

Focusing on the second transition, the results indicate two main variations when compared to the water system: (i) there is a slight increment of the 2nd CAC and a subsequent impediment on the aggregation for the system with 5 mM of NaH_2_PO_4_ and with the IFN-α 2b; and (ii) a decrease of the 2nd CAC for NaCl (pH = 7.3) and 150 mM of NaH_2_PO_4_ (pH = 7.4), indicating an intensification on the aggregation. The system with 25 mM KH_2_PO_4_ kept a value very close to the reference while the system with HCl does not show a 2nd CAC, as already mentioned. Finally, in the third transition, there is a decrease in FIL concentration for the systems with 25 mM of KH_2_PO_4_ (pH = 6.8) and 150 mM of NaH_2_PO_4_ (pH = 7.4), indicating that these two conditions boost the aggregation of [C_2_C_1_Im][C_4_F_9_SO_3_]. However, the remaining conditions do not show a significant variation compared to the system in water.

With the aim of obtaining additional information on the aggregation behaviour of the [C_2_C_1_Im][C_4_F_9_SO_3_], the degree of ionization of the aggregates, *α*, was calculated through the ratio of the slopes of the linear sections over and beneath each CAC. This parameter gives information on the packaging of the aggregates, meaning that a lower value of *α* indicates that the aggregate is more packed in its structure. From the parameter *α*, the degree of counterion binding, *β*, can be calculated. This parameter is associated with the charge density at the surface, the size, and the hydrophobic nature of the aggregate. It is given by the following equation:

*β* = 1 − *α* (1)

Thus, the lower the *α*, the higher the value of *β*, which is associated with a more packed structure. Then, the counterions will be associated and the polar counterparts will be more compacted due to the larger hydrophobic groups from the tensioactive anion. The *α* values of the systems studied in this work can be found in Table S1 of Supplementary Materials and Figure 1b, such as the ones obtained earlier for water [38]. Following the results represented in Figure 1b, for the 1st CAC, much lower values of *α* were found for the three simulated biological fluids when compared to the ones of water, supporting the results former reported. Therefore, these three conditions (NaCl, KH_2_PO_4_, and HCl) promote the aggregation behaviour of [C_2_C_1_Im][C_4_F_9_SO_3_] and result in aggregates more highly packed than the ones occurring in water. The opposite behaviour was found for the case of solutions with NaH_2_PO_4_ buffer and IFN-α 2b, where a slight increment of *α* was noticed. Therefore, the protein and the protein media hinder the aggregation of the FIL, resulting in aggregates with impaired packaging. Moreover, the concentration of NaH_2_PO_4_ does not affect the packaging of the aggregates, since the calculated *α* is the same. For the case of the 2nd CAC, the NaCl and KH_2_PO_4_ also show smaller *α* values compared to water, but this difference is not as strong as in the 1st CAC. No significant differences were found between water and the solutions in IFN-α 2b buffer and the one containing the protein. Finally, the 3rd CAC results show a more pronounced result for the case where the IFN-α 2b is present, where a higher value of *α* was found, indicating that the protein highly disrupts the packing of the aggregates formed in the 3rd CAC.

Another parameter was considered in this study related to the spontaneity of a surfactant to aggregate, given by the standard Gibbs free energy of aggregation, $\Delta G_{\mathrm{agg}}^{0}$. This parameter is provided by the pseudophase model of micellization [49], using the equation:

(2) $$\Delta G_{\mathrm{agg}}^{0}=RT\left(1+\beta \right)\ln x_{\mathrm{CAC}}$$

where *R* is the universal gas constant, *T* is the absolute temperature and $x_{\mathrm{CAC}}$ corresponds to the value of CAC in molar fraction. As long as this parameter is more negative, this means that the process of aggregation in the specific CACs is more spontaneous. The values of$\;\Delta G_{\mathrm{agg}}^{0}$ are illustrated in Table S1 of Supplementary Materials and Figure 1c. As expected, the values of the systems with simulated fluids for the case of the 1st CAC are much more negative compared with the reference, supporting the previous conclusions. No significant differences are denoted for the remaining systems. For the second transition, the variations are very small, meaning that the energy spent in the process of aggregation is not highly influenced by the tested conditions. Similar results are found for the 3rd CAC, excluding the case where the protein is in solution, where the value becomes slightly more positive, indicating an impairment of the FIL aggregation, as previously reported.

In conclusion, the results indicate that the simulated biological fluids (representing the bloodstream strength, gastric and intestinal fluid) improve the aggregation of [C_2_C_1_Im][C_4_F_9_SO_3_] because the 1st CAC occurs in a lower concentration of FIL, the structure of the aggregates is more packed, and the process of aggregation is more spontaneous when compared with the water + FIL system. This behaviour may be a result of the interactions between the FIL and the ions, increasing the ionic strength of the solution and forcing the molecules of FIL to aggregate sooner. Then, it will allow a reduced amount of FIL to be used in the formulation of the drug delivery systems once the aggregation is not impaired in the conditions associated with the routes of administration. Considering the routes of the administration represented by these simulated biological fluids (oral administration (intestinal fluid simulated by 25 mM KH_2_PO_4_ at pH 6.8 and gastric fluids simulated by 100 mM HCl at pH =1.2) and intravenous administration (bloodstream fluid simulated by 150 mM of NaCl at pH = 7.3)), a preferential route for the delivery of proteins with this FIL can be chosen. A drug administrated by the oral route must surpass the gastrointestinal tract until reaching the small intestine, where the drug will be absorbed into the liver, and finally enter the bloodstream to be distributed to its site of action, resulting in a lower bioavailability [50]. Consequently, a drug administrated via an intravenous system has full bioavailability since the absorption phase is skipped and lower doses are needed to have a therapeutic effect [50]. Therefore, similar results for the three simulated biological fluids lead to the conclusion that an intravenous administration will be more advantageous to FIL-based delivery systems. On the other hand, no significant influence was found in the case of the system with IFN-α 2b for the 1st and 2nd CAC. Only in the 3rd CAC is denoted an impairment of the aggregation by the presence of the protein, which might be a result of the protein-FIL interactions or the accommodation of IFN-α 2b in the [C_2_C_1_Im][C_4_F_9_SO_3_] aggregates.

#### 3.1.2. Surface Properties

For further investigation on the influence of the IFN-α 2b in the aggregation behaviour of FILs, the surface properties of [C_2_C_1_Im][C_4_F_9_SO_3_] were determined through two methods: (i) measurement of the surface tension and (ii) determination of the contact angles. The surface tension allows for the evaluation of the surface activity of the FIL in aqueous solutions. The common behaviour of the surface activity on SAILs is ruled by the adsorption at the air/water interface which reduces the interface energy between both phases [51]. Therefore, the surface tension decreases upon the addition of FIL up to a point where the accumulation of molecules at the interface is completed [51]. This breakpoint is known as the CMC, representing the formation of aggregates. Above the CMC, the value of the surface tension is kept constant due to the formation of more aggregates without surface activity. For the case of [C_2_C_1_Im][C_4_F_9_SO_3_], this behaviour was previously found in water, where only one transition, on the surface tension *versus* FIL concentration, was found. In these systems, the perfluoroalkyl chain points towards the inside of the aggregates, keeping the polar functional groups interacting with water [38].

**Figure 2:**
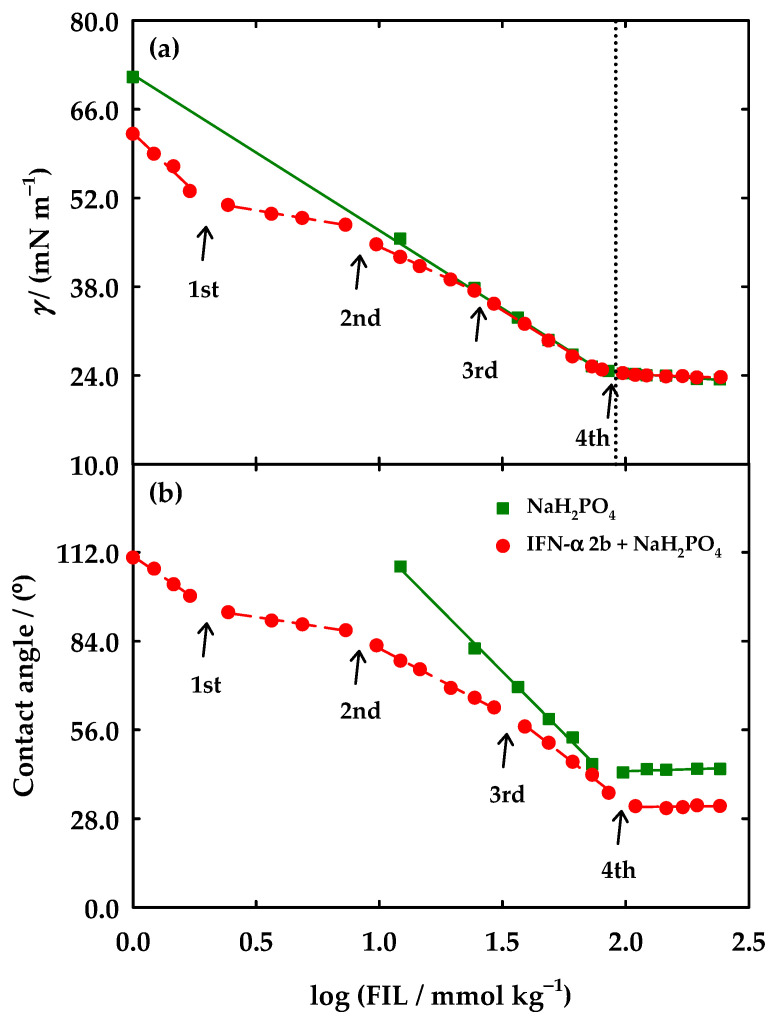
(**a**) Surface tension and (**b**) contact angles determined at 25 °C of [C_2_C_1_Im][C_4_F_9_SO_3_] aqueous solution in 5 mM NaH_2_PO_4_ (pH = 7.4) (green, 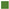) and with 5 μg/mL IFN-α 2b in 5 mM NaH_2_PO_4_ (pH = 7.4) (red, 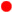). The vertical dotted line in (**a**) represents the value of CAC determined by surface tension in water on Langmuir 2015, 31, 1283–1295 [38]. The solid and dashed lines represent the fittings to obtain the value of FIL concentration where several breakpoints occurred.

In this work, several independent solutions of [C_2_C_1_Im][C_4_F_9_SO_3_] in 5 mM of NaH_2_PO_4_ at pH = 7.4 were prepared and the surface tension was measured. Afterwards, the addition of IFN-α 2b (with a fixed concentration of 5 μg/mL) was carried out for the same systems. To measure the surface tension, the density of the [C_2_C_1_Im][C_4_F_9_SO_3_] aqueous solutions in water at 25°C was obtained for the diluted region, and the values are described in Table S2 of Supplementary Materials. The density in a range of higher FIL concentrations was previously reported [38] and is also presented in Table S2 of Supplementary Materials. Figure 2a represents the results for both systems and an indication of the high purity of the studied solutions is revealed by the lack of a minimum around the breakpoints [51]. In the case of the system in 5 mM of NaH_2_PO_4_ (pH = 7.4), a very similar behaviour was found when compared with the results previously obtained for [C_2_C_1_Im][C_4_F_9_SO_3_] in water [38]. Only one breakpoint was observed, and the CAC and surface tension ($\gamma_{\mathrm{CAC}}$) values were determined by the linear fitting of the points after and before the breakpoint. This behaviour is very similar to the one of water (see dotted line in Figure 2a and Table S3 of Supplementary Materials). The transitions corresponding to the 1st and 2nd CAC obtained by conductometric titration are not found in the surface tension profile, while the observed discontinuity reveals a CAC value (81.59 mmol·kg^−1^) very close to that of the 3rd CAC (80.35 mmol·kg^−1^), obtained by conductometric titration (see Tables S1 and S3 of Supplementary Materials).

The surprising behaviour is found in the system of the [C_2_C_1_Im][C_4_F_9_SO_3_] with the IFN-α 2b. Four breakpoints were found, as observed in Figure 2a. The CAC and respective $\gamma_{\mathrm{CAC}}$ values are in Table S3 of Supplementary Materials. Three additional transitions occur when compared with the system without the protein, indicating that the IFN-α 2b has a much more active role in the surface activity of the FIL. The first breakpoint occurs at a concentration eight times lower than the 1st CAC, determined by conductometric titration. The second and third transitions (6.105 and 21.61 mmol·kg^−1^, respectively) have values about two times smaller when compared with the 1st and 2nd CAC (15.19 and 43.57 mmol·kg^−1^, respectively). The fourth breakpoint has a similar value to the 3rd CAC of the FIL (81.59 and 83.44 mmol·kg^−1^, respectively) as well as the CAC and $\gamma_{\mathrm{CAC}}$ values, being close to the ones found for the system without protein. These results indicate that the IFN-α 2b decreases the surface tension of the system and promotes the formation of different FIL aggregates at the interface of air/water at a lower FIL concentration. The high influence of the protein on the surface activity of the FILs made the method much more sensitive to alterations in the conformation of the FILs aggregates.

After the evaluation of the CACs, other properties can be taken from the surface tension data. Information on the adsorption occurring in the air/water interface can be determined from the maximum surface excess concentration, $\Gamma_{\max}$, which is given by the Gibbs adsorption isotherm [52]:

(3) $$\Gamma_{\max}=-\frac{1}{2.303nRT}{\left(\frac{d\gamma }{\mathrm{dlog}C}\right)}_{T}$$

where *n*, *R*, *T,* and *C* are the number of species in solution, the universal gas constant, the absolute temperature, and the concentration of FIL, respectively. The *n* parameter is deduced from the degree of ionization of aggregates, α, as *n* = 2 − α, determined from the conductivity profile of the FIL and assumed to be the same for the surface layer in ionic surfactants [53]. The α value of the 3rd CAC was used to calculate the $\Gamma_{\max}$ of the only transition occurring in [C_2_C_1_Im][C_4_F_9_SO_3_] in buffer and the fourth breakpoint of the solution with protein. The values of $\Gamma_{\max}$ are expressed in Table S3 of Supplementary Materials, as well as the ones of water for comparison purposes. The $\Gamma_{\max}$ of [C_2_C_1_Im][C_4_F_9_SO_3_] has the lowest value for the water system, followed by the buffer and finally the solution with protein.

From the $\Gamma_{\max}$ and Avogadro’s number (*N*_A_) the minimum area occupied per surfactant molecule at the air/water interface, *A*_mim_, can be obtained as set by:

(4) $$A_{\min}=\frac{10^{18}}{N_{A}\Gamma_{\max}}$$

Therefore, the bigger the $\Gamma_{\max}$, the less is *A*_mim_, and the surfactant molecules are rearranged in a denser structure at the solution surface [54]. Therefore, looking for the values of both parameters in Table S3 of Supplementary Materials, the [C_2_C_1_Im][C_4_F_9_SO_3_] assumes a denser arrangement at the surface in the presence of the protein, given by the highest value of $\Gamma_{\max}$ and lowest value of *A*_mim_.

In addition, the parameter that explains the efficacy of a surfactant to reduce the surface tension, Π_CAC_, was determined, expressing the maximum surface tension diminution resulting from the dissolution of surfactant molecules. This parameter is determined by the surface tension of the solvent ($\gamma_{0}$) and the $\gamma_{\mathrm{CAC}}$, as follows:

(5) $$\Pi_{\mathrm{CAC}}=\gamma_{0}-\gamma_{\mathrm{CAC}}$$

Them, from Π_CAC_, the standard free energy of adsorption, $\Delta G_{\mathrm{ad}}^{0}$, was calculated using the following equation [55]:

(6) $$\Delta G_{\mathrm{ad}}^{0}=\Delta G_{\mathrm{agg}}^{0}-\frac{\Pi_{\mathrm{CAC}}}{\Gamma_{\max}}$$

The results of Π_CAC_ and$\;\Delta G_{\mathrm{ad}}^{0}$ are also reported in Table S3 of Supplementary Materials. When comparing the values of $\;\Delta G_{\mathrm{ad}}^{0}$ with the ones of $\;\Delta G_{\mathrm{agg}}^{0}$, it is clear that the process of adsorption of the surfactant in the air/water interface is more spontaneous that the process of aggregation due to the more negative values (see Tables S1 and S3 of Supplementary Materials) for the three systems (water, buffer, protein). Moreover, the previous results are endorsed by the $\;\Delta G_{\mathrm{ad}}^{0}$ parameter, since the less negative value is found for the case of [C_2_C_1_Im][C_4_F_9_SO_3_] with IFN-α 2b, indicating that the protein is also challenging for the adsorption of the FIL in the interface.

Finally, aiming to disclose information on the self-aggregation process and the shape of the aggregates, the critical packing parameter, *P*, was also calculated in this work, as follows [56]:

(7) $$P=\frac{V_{0}}{A_{\min}l_{c}}$$

where *V*_0_ and *l*_c_ are the volume occupied by the hydrophobic chains in the aggregate core and the critical chain length, respectively. The structure of the aggregates is defined as spherical when *P* ≤ 0.33, cylindrical for *P* ≤ 0.5, lamellar when *P* ≤ 1, and inverted for *P* > 1 [38]. In summary, a modification of the Tanford equations [57] was used for fluorinated compounds and the critical packing parameter was calculated through the following equations:

(8) $$V_{0}\left({\mathrm{nm}}^{3}\right)=0.0545+0.0380\left(n_{c}-1\right);\;l_{c}\left(\mathrm{nm}\right)=0.200+0.134\left(n_{c}-1\right)$$

(9) $$V_{0}\left({\mathrm{nm}}^{3}\right)=0.0424+0.0416\left(n_{c}-1\right);\;l_{c}\left(\mathrm{nm}\right)=0.204+0.130\left(n_{c}-1\right)$$

where the *n*_c_ is the number of carbon atoms in the perfluoroalkyl side chain of the anion. The *P* values obtained for each system are represented in Table S3 of Supplementary Materials. Analysing the data, the aggregates of [C_2_C_1_Im][C_4_F_9_SO_3_] assume different conformations in the three systems. For the case of water, the aggregates have a cylindrical shape (0.33 < *P* ≤ 0.5). The aggregates of the FIL in 5 mM NaH_2_PO_4_ (pH = 7.4) have a *P* very close to 0.5, which is characteristic of cylindrical and lamellar aggregates. The presence of the protein increases the *P* to values close to 0.7, indicating a lamellar structure of the aggregates. This behaviour might indicate that the aggregates rearrange their structure to accommodate the protein or that the interactions between FIL and IFN-α 2b induce these changes.

A study of the contact angles was also carried out in this work. The contact angles are related to the wettability of a surface-active liquid on a solid surface. When a drop of a liquid is placed on a solid surface, the shape of the drop results from the effect of the interfacial tensions between solid (*s*) − water (*w*) − air (*a*). These interfacial tensions are related to the contact angles (*θ*) by Young’s equation [51]:

(10) $$\gamma_{s/a}=\gamma_{s/w}+\gamma_{w/a}\cos\theta$$

When the drop of a liquid has a contact angle below 90° with the solid, it is established that the liquid wets the surface and is categorized as hydrophilic (e.g., water). When the angle is superior to 90°, the liquid is considered hydrophobic and does not wet the solid (e.g., oil) [51]. Figure 2b shows the contact angles calculated for the systems of [C_2_C_1_Im][C_4_F_9_SO_3_] in 5 mM NaH_2_PO_4_ (pH = 7.4) and with 5 μg/mL IFN-α 2b in 5 mM NaH_2_PO_4_ (pH = 7.4). A more visual scheme of the behaviour of the drops of each measurement can be found in Figures S2 and S3 of Supplementary Materials. The results support the behaviour found for the surface tension measurements. The value of FIL concentration in the breakpoint found for the contact angles (80.27 mmol⸱kg^−1^) is very close to the one obtained through the surface tension (80.35 mmol⸱kg^−1^) (see Table S3 of Supplementary Materials). For the case of [C_2_C_1_Im][C_4_F_9_SO_3_] with 5 μg/mL IFN-α 2b system, four breakpoints were also found, where the first and second transitions have very similar values to the ones obtained with γ, while the third (21.61 (*γ*) and 38.18 (*θ*) mmol⸱kg^−1^) and fourth (81.59 (*γ*) and 104.7 (*θ*) mmol⸱kg^−1^) transitions occurred at a higher FIL concentration (see Table S3 of Supplementary Materials). In addition, Figures S2 and S3 of Supplementary Materials show a clear decrease in the contact angles with the solid surface with the increment in the FIL concentration. Therefore, the surfactant nature of FILs is characterized by increased wettability.

#### 3.1.3. Characterization of the Self-Assembled Aggregates

**Figure 3:**
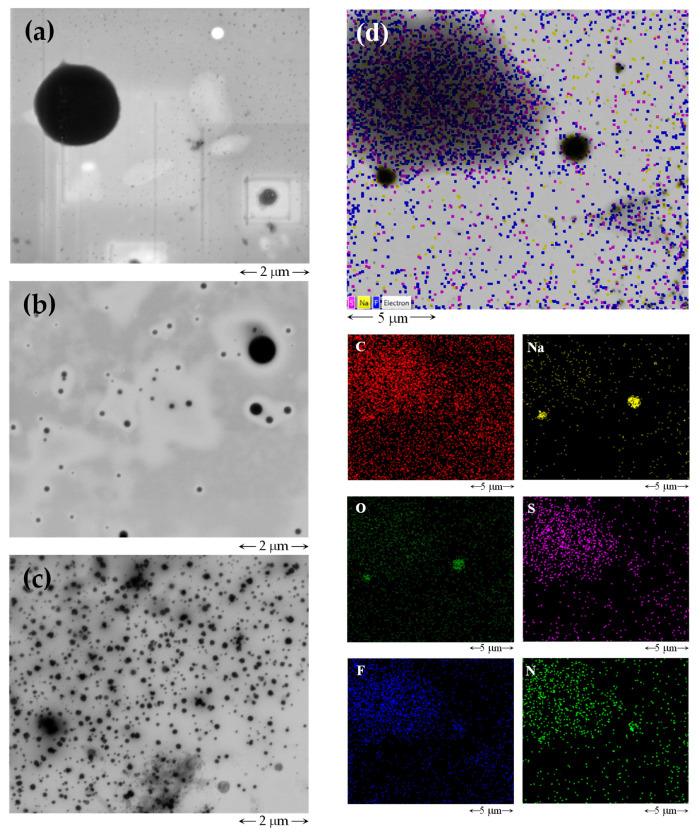
TEM images of (**a**) IFN-α 2b at 0.0001 μg/mL; (**b**) [C_2_C_1_Im][C_4_F_9_SO_3_] at 29.2 mM; and (**c**) [C_2_C_1_Im][C_4_F_9_SO_3_] at 29.2 mM in the presence of IFN-α 2b at 0.0001 μg/mL. (**d**) EDS analysis of [C_2_C_1_Im][C_4_F_9_SO_3_] with IFN-α 2b at the same conditions. All samples were prepared in 150 mM NaH_2_PO_4_ (pH = 7.4) and measured at 25 °C.

Once the formation of FILs aggregates was determined in the presence of the protein, the characterization of those aggregates was under investigation. First, scanning transmission electron microscopy (STEM) with energy-dispersive X-ray spectroscopy (EDS) was performed to have insight into the size and morphology of [C_2_C_1_Im][C_4_F_9_SO_3_] aggregates and the impact of IFN-α 2b on them. A concentration above the 1st CAC (approximately two times) of [C_2_C_1_Im][C_4_F_9_SO_3_] was selected to ensure the formation of FIL aggregates. Three different solutions were prepared: (i) IFN-α 2b at 0.0001 μg/mL; (ii) [C_2_C_1_Im][C_4_F_9_SO_3_] at 29.2 mM; and (iii) [C_2_C_1_Im][C_4_F_9_SO_3_] (29.2 mM) with IFN-α 2b (0.0001 μg/mL). The three samples were prepared in 150 mM of NaH_2_PO_4_ (pH = 7.4). Figure 3 shows the TEM images obtained for the three solutions on two different scales. The TEM image of IFN-α 2b is illustrated in Figure 3a and large dark circles are observed with a size up to around 2 μm, which can indicate that the protein forms large aggregates. In the case of the FIL, Figure 3b shows considerably smaller dark circles with sizes around 0.05 to 0.7 μm. A comparison with the TEM images of [C_2_C_1_Im][C_4_F_9_SO_3_] in water (aggregates with sizes around 0.1 to 0.2 μm) [38] shows that the addition of the buffer as dispersant also has an impact on the FIL aggregates, with the formation of bigger ones (up to 0.7 μm). These results suggest that the nature of the dispersant plays an important role in the stabilization of the self-assembled structures, with an increment in the size in the presence of a buffer. The solution with [C_2_C_1_Im][C_4_F_9_SO_3_] and IFN-α 2b (Figure 3c) presents smaller dark circles (around 0.1 to 0.5 μm), and the morphology of the circles seems to change and become more irregular than the circles in the pure compounds. Therefore, an interaction between the FIL and the protein might justify these differences in the size and morphology of the aggregates. This strong interaction between the aggregates of the FIL and the protein can produce complexation between the FIL and the protein, forming aggregates with irregular shapes. This interaction may have positive effects on the stabilization or dispersion of the aggregates of the biomolecule. However, encapsulation of the protein cannot be confirmed by these images.

Moreover, the samples were subject to EDS analysis which allowed the elemental analysis of the aggregates, providing images of the distribution of each element present in the aggregate. The IFN-α 2b is constituted by carbon, hydrogen, nitrogen, oxygen, and sulphur, the buffer elements are explicit in its nomenclature, NaH_2_PO_4_, and the FIL is composed of carbon, hydrogen, nitrogen, sulphur, and fluorine elements. The EDS analysis of IFN-α 2b can be found in Figure S4 of Supplementary Materials and the elements, sodium, oxygen and phosphorus, were identified in this solution. The EDS images lead to the conclusion that these elements, predominant in the buffer structure, are in the darker zones that correspond to the IFN-α 2b aggregates. Then, IFN-α 2b aggregates in a self-assembled structure where an accumulation of the elements of the buffer is identified. There is also the presence of several smaller circles where the buffer elements are concentrated that may be the molecules of protein that do not aggregate or are in a different conformation. Figure S5 of Supplementary Materials shows the same analysis for the [C_2_C_1_Im][C_4_F_9_SO_3_] solution. In this case, as expected, the sulphur and fluorine elements of the anion are identified in the areas where the dark circles of FILs aggregates were found. Moreover, the elements of buffer, such as sodium and oxygen (from FIL and buffer), are more concentrated in the FIL aggregates, leading to the conclusion of a strong effect of the buffer in the FILs aggregates. The EDS analysis of the mixture [C_2_C_1_Im][C_4_F_9_SO_3_] with IFN-α 2b is illustrated in Figure 3d and the results show that the irregular dark circles have a composition similar to both protein and FIL, with a diffusion of the elements of the buffer and the FIL in the dark spots. In this case, it was also possible to obtain nitrogen analysis due to the higher population of this element in the solution (from the protein and FIL). This element is also concentrated in the area that corresponds to the aggregates found in the images. These results support a protein-FIL interaction due to the dispersion of the components of both species focused on the dark spots. Therefore, the dark circles correspond to an aggregate formed by the FIL and the protein, which allows the dispersion of the bigger aggregates formed by the isolated protein.

**Figure 4:**
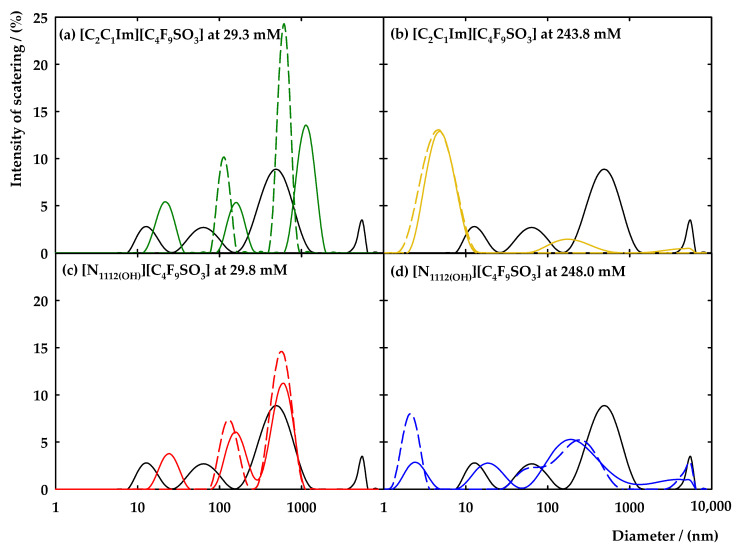
DLS spectra of IFN-α 2b at 50 μg/mL (black solid line) with [C_2_C_1_Im][C_4_F_9_SO_3_] at (**a**) 29.3 mM and (**b**) 243.8 mM; and [N_1112(OH)_][C_4_F_9_SO_3_] at (**c**) 29.8 mM and (**d**) 248.0 mM. Solid lines represent the samples with protein and FIL and the dashed lines illustrate the FILs blanks. All samples were prepared in 150 mM NaH_2_PO_4_ at pH = 7.4 and measured at 25 °C.

Dynamic light scattering (DLS) was carried out in this work to further characterize the aggregates of FIL in the presence of the protein IFN-α 2b. This is a useful tool with a non-invasive character that gains access to information on the size of the aggregates. For that, two concentrations of [C_2_C_1_Im][C_4_F_9_SO_3_] (two times the 1st CAC and above the 3rd CAC) were selected, aiming to understand how the aggregates of the FIL are impacted by the protein. Figure 4a,b shows the DLS spectra of [C_2_C_1_Im][C_4_F_9_SO_3_] at 29.3 mM and 243.8 mM, respectively, in 150 mM NaH_2_PO_4_ at pH = 7.4 with IFN-α 2b at 50 μg/mL. The FIL blanks in each studied concentration and the protein blank are also included in Figure 4a,b. The characteristic spectrum of the IFN-α 2b (black solid line of Figure 4) comprises four characteristic peaks that correspond to aggregates of sizes between 8 and 25 nm, 28 and 145 nm, 180 and 1400 nm, and 4000 and 6800 nm. The structural elucidation of the interferon-alpha 2b revealed that this protein exists in crystals as a dimer in the native state. The biological role of the dimer is not fully comprehended, and the literature suggests that this protein is active in a monomer conformation. However, the dimer was associated with a possible inactive storage conformation of the molecule or may have relevance in the biological activity of the protein, once the dimerization is relevant in other types of interferons [58]. The [C_2_C_1_Im][C_4_F_9_SO_3_] shows two peaks around 80 to 170 nm and 430 to 1000 nm in the concentration, two times the 1st CAC and one peak between 2 and 14 nm for the concentration above the 3rd CAC (Figure 4b). For the samples containing both FIL and IFN-α 2b, the characteristic peaks of the FIL slightly shifted to bigger values (around 95 to 270 nm and 620 to 1970 nm in Figure 4a). A new peak appears on the spectrum, between 10 and 40 nm, that may correspond to smaller aggregates typical of the protein, with a slight shift in the hydrodynamic diameter. Figure 4b shows that in the mixture of FIL and protein, the characteristic peak of the [C_2_C_1_Im][C_4_F_9_SO_3_] is slightly tightened and two new peaks appear on the spectrum between 60 and 615 nm and 2000 and 6200 nm with a very reduced intensity. These new peaks can be associated with the aggregates of the protein either in solution or to another conformation that is a result of the interaction of the FIL with the protein. Therefore, these results endorse those previously obtained, where strong interactions between the FIL and the IFN-α 2b occur and have an effect on the aggregates of the FIL.

Aiming to seek more evidence on the FIL aggregates’ behaviour and explore the possibility of encapsulating the IFN-α 2b with those aggregates, [N_1112(OH_][C_4_F_9_SO_3_] was also studied by DLS. In recent work, preliminary results pointed out the [N_1112(OH_]^+^ cation as a promotor of interactions between FILs and IFN-α 2b [46]. Therefore, [N_1112(OH_][C_4_F_9_SO_3_] concentrations of 29.8 mM and 248 mM were selected to ensure aggregation of this FIL and the results are depicted in Figure 4c,d. In the case of a concentration two times greater than the 1st CAC (29.8 mM, Figure 4c), two characteristic peaks are found, approximately from 70 to 250 and 290 to 1080 nm, whereas for the case of a concentration above the 3rd CAC (248 mM, Figure 4d), three peaks were recorded between 1 and 4 nm, 28 and 825 nm, and 2800 and 6440 nm. Analysing the mixture of [N_1112(OH_][C_4_F_9_SO_3_] with IFN-α 2b (see Figure 4c), the peaks of the FIL have a diminished intensity and there is a small shift of the first peak to values of 85 to 280 nm. Another peak arises in the range of 13 to 44 nm, which does not overlap either with the peaks of the protein or the FIL. However, it might result from the smaller aggregates of the IFN-α 2b that have a change in their hydrodynamic diameter. Figure 4d also shows that the intensity of the peak of the smaller aggregates of FIL have a decreased intensity and the intermediate peak had suffered a deconvolution in two peaks, shifting to lower values of hydrodynamic diameter. Therefore, an interaction between protein-FIL might be occurring, leading to modifications in the aggregates of FILs. The results are very similar to the ones obtained with [C_2_C_1_Im][C_4_F_9_SO_3_] (see Figure 4a,c), leading to the conclusion that the aggregates of both FILs have very similar interactions with this protein. Therefore, the main structural feature of the FIL controlling this behaviour is the anion. Furthermore, these results support those previously obtained, indicating that the aggregation of the protein is reduced, and smaller aggregates are formed. The FIL aggregates do not completely cover the protein (encapsulation), but the DLS results confirm the strong interaction between FILs and IFN-α 2b.

Moreover, the polydispersity index (PdI) was obtained by DLS which determines the heterogeneity of the sample in the function of the size. International standards organizations (ISOs) are defined for the characterization of nanomaterials based on the PdI, where it is considered that a PdI < 0.05 characterizes monodisperse samples, whereas PdI > 0.7 is characteristic of the polydisperse distribution of the particles (ISO 22,412:2017) [59]. Table S4 of Supplementary Materials shows the PdI values obtained from the DLS spectra acquisition for each experiment. The analysis of the samples with [C_2_C_1_Im][C_4_F_9_SO_3_] demonstrates two distinct behaviours: (i) the presence of FIL two times the 1st CAC in the IFN-α 2b increases its polydispersity and decreases the PdI of the FIL aggregates; (ii) in the concentration above the 3rd CAC with IFN-α 2b, the polydispersity is diminished when related with the protein alone and increased for the FIL aggregates. In the case of the cholinium cation at lower concentrations, the behaviour is similar to the imidazolium cation. However, for the concentration above the 3rd CAC, the PdI of the solution FIL + protein is always higher than the protein itself and the FIL aggregates. This parameter supports the results previously discussed related to the size of the aggregates.

The overall conclusions of this section strengthen the hypothesis of very strong interactions occurring between the selected FILs and the protein IFN-α 2b. This protein has a strong impact on the FILs aggregates, leading to modifications in the aggregation process, surface activity, shape, size, and morphology of those aggregates. These results raise the assumption that a complexation between both molecules is happening and allows the FIL to disperse the aggregates of the protein by transporting the protein without fully covering the surface of the biomolecule.

### 3.2. Interactions of the Fluorinated Ionic Liquids with Interferon-Alpha 2b

The interactions between FILs and proteins are of great relevance to understanding the potential of FILs and their application as DDSs of biopharmaceuticals. The stability and activity of proteins depend on a delicate balance between the interactions of biomolecules with the surroundings, and the addition of FIL can completely alter their conformation and subsequently hinder their function. In this work, several efforts were carried out to strengthen knowledge of the interactions between FILs and IFN-α 2b. For that goal, both [C_2_C_1_Im][C_4_F_9_SO_3_] and [N_1112(OH_)][C_4_F_9_SO_3_] were selected in a concentration range where the aggregation behaviour plays the key role in their activity. Firstly, several spectroscopy methods were used to obtain information on protein-FIL interactions via insights into the structural conformation of the protein, such as UV–Vis, fluorescence, and circular dichroism spectroscopies. In the end, the binding affinity between the FILs and IFN-α 2b was accessed by performing microscale thermophoresis.

#### 3.2.1. Spectroscopy Analysis

As a first approach, UV–Vis spectrophotometry was used to measure the absorption spectra of the IFN-α 2b. This method follows the behaviour of the chromophores composing the structure of the proteins, such as the amide group of the protein backbone and the aromatic amino acids such as tryptophan, tyrosine, and phenylalanine [60]. Therefore, these structural features act as spectral probes of significant perturbations occurring in the local environment of the protein through their exposure to the solvent [60]. Thus, the IFN-α 2b absorption spectrum is very similar to that of most proteins, with a characteristic band between 260 and 300 nm. Any alteration on the profile of this band may indicate a perturbation of the protein chromophores, and consequently, the existence of an interaction with a ligand [40]. For example, the coverage of the protein surface by the FIL can be reflected in the absorption spectra by the absence of the characteristic absorption band or reflected in the turbidity of the solution due to the presence of protein-FIL aggregates [40].

**Figure 5:**
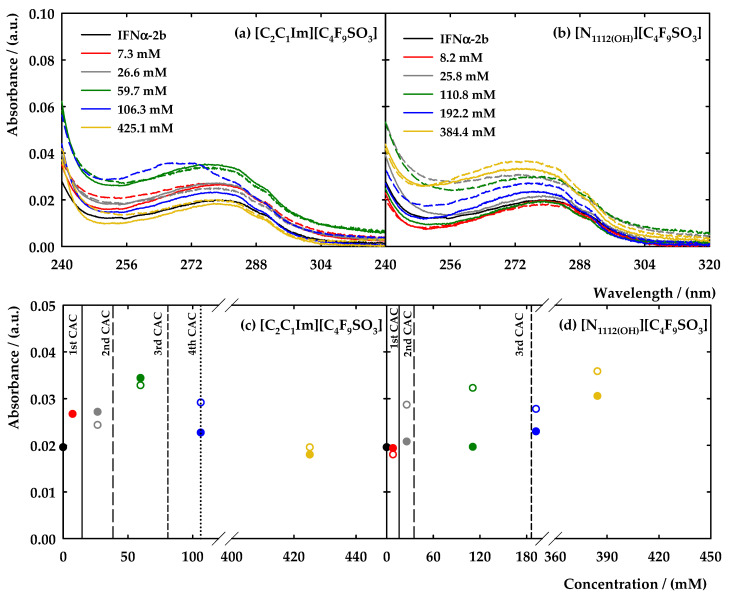
UV–Vis absorption spectra of (**a**) [C_2_C_1_Im][C_4_F_9_SO_3_] and (**b**) [N_1112(OH)_][C_4_F_9_SO_3_] with IFN-α 2b at 20 μg/mL (black solid line) at different concentrations. All samples were prepared in 5 mM NaH_2_PO_4_ (pH = 7.4) and measured at 25 °C. In (**c**,**d**) the variation in the absorbance at 280 nm is represented. The solid lines in (**a**,**b**) and full symbols in (**c**,**d**) correspond to the measurements at 0 h. The dashed lines in (**a**,**b**) and the empty symbols in (**c**,**d**) correspond to the measurements after 24 h of incubation. The colours of the symbols correspond to the colours of the lines.

Solutions of [C_2_C_1_Im][C_4_F_9_SO_3_] and [N_1112(OH)_][C_4_F_9_SO_3_] were prepared in five different concentrations to cover all the aggregation levels: (i) below the 1st CAC (7.3 mM for [C_2_C_1_Im]^+^ and 8.2 mM for [N_1112(OH)_]^+^) to understand the interplay of the FILs monomers with the protein; (ii) above the 1st CAC (26.6 mM for [C_2_C_1_Im]^+^ and 25.8 mM for [N_1112(OH)_]^+^); (iii) above the 2nd CAC (59.7 mM for [C_2_C_1_Im]^+^ and 110.8 mM for [N_1112(OH)_]^+^); (iv) above the 3rd CAC (106.3 mM for [C_2_C_1_Im]^+^ and 192.2 mM for [N_1112(OH)_]^+^); and (v) above the 4th CAC (425.1 mM for [C_2_C_1_Im]^+^ and 384.4 mM for [N_1112(OH)_]^+^ where the value of the 4th CAC of [C_2_C_1_Im][C_4_F_9_SO_3_] was used as a reference for both FILs). The solutions were prepared in 5 mM of NaH_2_PO_4_ (pH = 7.4) with 20 μg/mL of IFN-α 2b. The measurements were made after the preparation of the solutions (time 0 h) and after 24 h of incubation to increase the contact time between the aggregates of the FIL and the protein. The results can be found in Figure 5a,b along with the blank of the protein for comparison purposes. To ease the discussion of the results and the comparison between the studied conditions, the value of absorbance at a wavelength of 280 nm (characteristic band) is represented in Figure 5c,d.

Figure 5a,c show the results concerning [C_2_C_1_Im][C_4_F_9_SO_3_]. In almost all concentrations of FIL, a slight increase in the absorbance is perceived, when compared to the value of IFN-α 2b. However, this increment was not verified in the most concentrated solution of FIL. Additionally, the increment is not proportional to the FIL concentration, which may indicate that the different aggregates do not influence the surroundings of the protein in the same manner. The highest value of Abs_280nm_ is found for the FIL concentration above the 2nd CAC. Comparing the different times of incubation, the results indicate that only in the concentration above the 3rd CAC is there a significant difference in the value of Abs_280nm_, and a shift of the band towards low values of absorbance is visible. Hence, most differences are found in the case of the concentrations of FIL above the 2nd and 3rd CAC, emphasising that the aggregates of [C_2_C_1_Im][C_4_F_9_SO_3_] interact with the IFN-α 2b, boosting the exposition of the chromophores to the solvent. However, in any case, the characteristic band of the protein is reduced or omitted, indicating that the FIL does not cover the chromophores of the protein, and, consequently, there is no indication that the protein is being encapsulated by the [C_2_C_1_Im][C_4_F_9_SO_3_] aggregates.

Analysing the results for [N_1112(OH)_][C_4_F_9_SO_3_], Figure 5b,d elucidate the interactions with the IFN-α 2b. A [C_2_C_1_Im][C_4_F_9_SO_3_]-like behaviour is found, where the absorbance of the protein band increases in the presence of the FIL. However, in this case, it is proportional to the increment in FIL concentration and is much more evident after 24 h incubation. The most significant differences are found in the concentrations where the FIL aggregates are formed, reinforcing the assumption of an interaction between the [N_1112(OH)_][C_4_F_9_SO_3_] aggregates and protein. This interaction also promotes the disclosure of the chromophores to the solvent, increasing the absorbance intensity.

In summary, UV–Vis spectrophotometry supports the presence of an interaction between the FILs aggregates and the protein, augmenting the exposition of IFN-α 2b chromophores to the solvent. These results also indicate that [C_2_C_1_Im]^+^ and [N_1112(OH)_]^+^ cations present similar behaviour, supporting the anion having the most important role in this interaction. Comparing these results with the ones obtained for lysozyme [40], the protein-FILs interactions have high specificity and depend on the target protein. In this case, there were no significant alterations in the characteristic peak of IFN-α 2b, and the studied solutions do not present turbidity. The FILs aggregates do not seem able to encapsulate the protein, or at least, they do not hide the residues that are studied within this technique.

Another well-established analytical technique to study protein-FIL interactions is fluorescence spectroscopy. The intrinsic fluorescence of the proteins is an outcome of the aromatic residues, where usually the dominant fluorophore is the tryptophan [61]. As observed in the previous section, the tryptophan has an absorption of around 280 nm, and its emission is usually near 340 nm [61]. The emission spectrum of the tryptophan is very sensitive to solvent polarity. Therefore, a shift of the maximum emission towards smaller wavelengths, the so-called blue shift, indicates that the aromatic residue is concealed in the native structure of the protein [61]. A shift of the emission spectrum to longer wavelength values, known as the red shift, indicates an exposition of the tryptophan and is associated with the unfolded state of the protein structure [61]. Consequently, a blue shift of the emission is associated with a productive effect on the structure of the protein, whereas a red shift indicates that the ligand has a destructive impact on the protein’s structural features. Additionally, the increase or decrease in the intensity of the emission spectra is related to the amount of exposition of the fluorophores to the solvent.

In this work, the emission spectrum of the IFN-α 2b was obtained in a range of 300 to 450 nm as well as a maximum emission of around 340 nm (Figure S6 of Supplementary Materials). The sample was excited at 280 nm to obtain the maximum emission of the intrinsic fluorescence of the protein. Typically, an excitation of 295 nm is used to selectively excite the tryptophan and avoid the response of other aromatic residues such as tyrosine [61]. However, in this case, the goal was to obtain a maximized emission spectrum to avoid issues related to signal intensity and gather information on the impact and interactions of FIL aggregates with IFN-α 2b [58].

**Figure 6:**
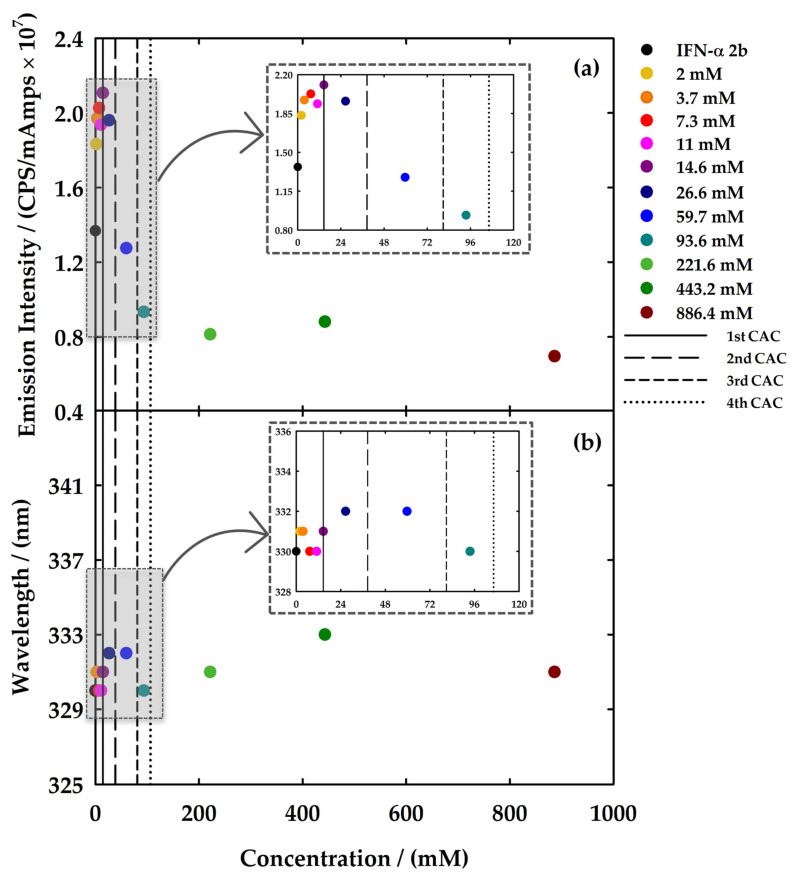
(**a**) Emission intensity and (**b**) wavelength variation of the maximum of the emission spectra of different concentrations of [C_2_C_1_Im][C_4_F_9_SO_3_] with IFN-α 2b at 20 μg/mL recorded at 280 nm. All samples were prepared in 5 mM NaH_2_PO_4_ (pH = 7.4) and measured at 25 °C.

**Figure 7:**
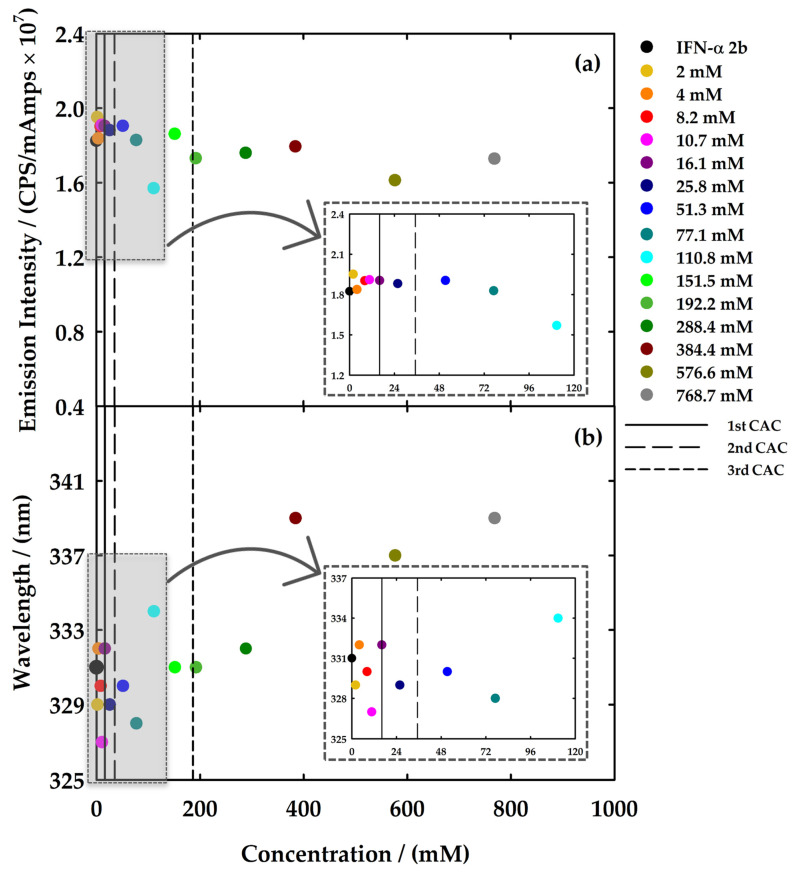
(**a**) Emission intensity and (**b**) wavelength variation of the maximum of the emission spectra of different concentrations of [N_1112(OH)_][C_4_F_9_SO_3_] with IFN-α 2b at 20 μg/mL recorded at 280 nm. All samples were prepared in 5 mM NaH_2_PO_4_ (pH = 7.4) and measured at 25 °C.

Both [C_2_C_1_Im][C_4_F_9_SO_3_] and [N_1112(OH)_][C_4_F_9_SO_3_] were selected to study the interactions with IFN-α 2b by fluorescence spectroscopy. Different solutions were prepared and measured in a range of concentrations covering all the aggregation steps of the FIL: (i) four samples below the 1st CAC to seek for interactions between the FILs monomers and the protein; (ii) two samples near and above the 1st CAC; (iii) one above the 2nd CAC; (iv) one above the 3rd CAC; and (v) three above the 4th CAC for [C_2_C_1_Im][C_4_F_9_SO_3_]. In the case of [N_1112(OH)_][C_4_F_9_SO_3_], additional samples between the 2nd and 3rd CAC and above the 3rd CAC were measured. The protein concentration was fixed to 20 μg/mL in all solutions. The emission spectra of all the measured solutions are illustrated in Figure S6 of Supplementary Materials for [C_2_C_1_Im][C_4_F_9_SO_3_] and [N_1112(OH)_][C_4_F_9_SO_3_]. To facilitate discussion of the results, the values of maximum emission intensity and the corresponding wavelength (*λ*_max_) are represented in Figure 6 and Figure 7 to provide information on the exposition of fluorophores of the protein and which type of interaction the ligands have with the protein, respectively.

Figure 6 corresponds to the results related to the impact of the variation in the concentration of [C_2_C_1_Im][C_4_F_9_SO_3_] in the protein emission. In this plot, two main comportments are observed concerning the intensity of the emission: the presence of FIL in concentrations under the 1st CAC increases the emission intensity of the IFN-α 2b. After the 1st CAC, a decrease of that intensity to values lower than the spectrum of IFN-α 2b is observed when the FIL concentration is higher. Figure 6b shows that in the range of concentrations below the 1st CAC, the variations in *λ*_max_ are between 0 to 1 nm (blue and red shift), and above the 1st CAC are found variations between 0 to 3 nm by a red shift. The results indicate that two different interactions are occurring within this system: (i) first, the FIL monomers interact with the protein and the fluorophores are more exposed to the solvent; and (ii) the aggregation of the FIL induces a quenching of the emission intensity, which might indicate that the interaction with the protein conceals the fluorophores from the solvent. The red shift of *λ*_max_ does not appear significant, and this FIL does not show a destructive behaviour towards the protein in this range of concentrations.

Figure 7 reports the results regarding the [N_1112(OH)_][C_4_F_9_SO_3_] and its influence on the IFN-α 2b emission. In Figure 7a, a distinct behaviour of the [C_2_C_1_Im][C_4_F_9_SO_3_] is noticed. Until the concentration above the 2nd CAC, the FIL does not show a considerable influence on the intensity of the emission of IFN-α 2b. Then, there is a slight decrease in the intensity in the concentrations close to the 3rd CAC, and after this transition, the intensity stabilizes once again until the concentration of 384 mM. These variations are very small when compared with the pronounced variations found for the emission intensity of the solutions with [C_2_C_1_Im][C_4_F_9_SO_3_] (Figure 6a). However, this comportment is not reflected in Figure 7b, where much greater differences are found in the *λ*_max_. The variation of *λ*_max_ in the concentrations above the 1st CAC are within 0 and 4 nm and mostly in a blue shift regime, indicating constructive interactions between the [N_1112(OH)_][C_4_F_9_SO_3_] and IFN-α 2b. This behaviour is conserved until 77 mM (above the 2nd CAC), meaning that the FILs aggregates also have a positive interaction with the protein, making its fluorophores assume a tighter conformation regarding the solvent. After that, there is a red shift of 3 nm in the concentration close to the 3rd CAC, followed by a stabilization of *λ*_max_. Finally, in concentrations above 384 mM, there is an interaction with the protein dictated by a red shifting up to 8 nm, meaning that unfolding of the protein might be occurring, completely exposing the fluorophores to the solvent. To sum up, the [N_1112(OH)_][C_4_F_9_SO_3_] has a very ambiguous behaviour, since in concentrations below the 3rd CAC, the interactions favour the structure of the protein for a more closed conformation. Between 288 and 384 mM, there is a complete alteration of the behaviour, and the FIL aggregates assume destructive interactions with the protein, leading to its unfolding. Nevertheless, these FIL concentrations are in a much higher range than the ones that must be used in a biomedical approach, and this result does not affect the applicability of this FIL.

Analysing both systems, a decrease in the intensity in the region between ~14 mM and ~110 mM (above the 1st CAC) was found, which was more pronounced for [C_2_C_1_Im][C_4_F_9_SO_3_], which is called the quenching effect. A very well-known equation, the Stern–Volmer, was used to characterize the quenching occurring in this region. This equation characterizes the collisional quenching that can occur when the excited state of the fluorophore is disabled due to contact with another molecule (quencher), without chemical alteration [61]. Therefore, the collisional quenching is described by:

(11) $$F_{0}/F=1+K\left[Q\right]=1+k_{q}\tau_{0}\left[Q\right]$$

where *F*_0_ and *F* are the fluorescence intensities of the fluorophore in the absence and presence of the quencher, respectively, *K* is the Stern–Volmer quenching constant, *k*_q_ is the biomolecular quenching constant, *τ*_0_ is the unquenched lifetime, and [Q] is the quencher concentration. The *K* constants were obtained through the slope of the $F_{0}/F\;$*versus* the quencher concentration, yielding a *K* of 1.06 × 10^−2^ mM^−1^ for [C_2_C_1_Im][C_4_F_9_SO_3_] and 1.95 × 10^−3^ mM^−1^ for [N_1112(OH)_][C_4_F_9_SO_3_]. This value gives information on the sensitivity of the fluorophore to a quencher. The higher value of *K* can indicate that the [C_2_C_1_Im][C_4_F_9_SO_3_] is freer in solution or at the surface of the biomolecule. The lower value of [N_1112(OH)_][C_4_F_9_SO_3_] can be related to the FIL being more buried in the macromolecule, which is in concordance with the disruption of the protein conformation at higher FIL concentrations. Through the $\tau_{0}$ of IFN-α 2b (approximately 2.95 × 10^−9^ s [62]), it was possible to obtain the *k*_q,_ considering that:

(12) $$K\left[Q\right]=k_{q}\tau_{0}\left[Q\right]$$

For [C_2_C_1_Im][C_4_F_9_SO_3_], the value of *k*_q_ is 3.59 × 10^6^ mM^−1^s^−1^ for [C_2_C_1_Im]^+^ and 6.61 × 10^5^ mM^−1^s^−1^ for [N_1112(OH)_]^+^. The comparison of these values with the largest possible biomolecular quenching constant for dynamic collision, 2.0 × 10^7^ mM^−1^s^−1^ [63], indicates that the quenching is dynamic, and no ground-state complex is formed during the quenching process.

**Figure 8:**
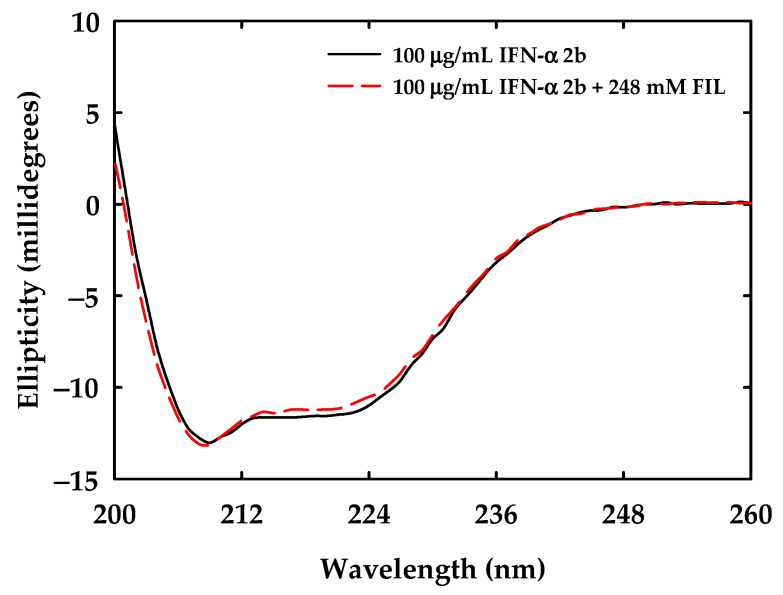
CD spectra of [N_1112(OH)_][C_4_F_9_SO_3_] at 248 mM in the presence of IFN-α 2b at 100 μg/mL in 150 mM NaH_2_PO_4_ (pH = 7.4) and measured at 25 °C.

Circular dichroism (CD) spectroscopy is the most conventional method used to promptly assess the secondary structure of proteins [64]. In this work, it was used to infer the changes in the secondary structure of IFN-α 2b upon the addition of [N_1112(OH)_][C_4_F_9_SO_3_]. The CD spectrum for [C_2_C_1_Im][C_4_F_9_SO_3_] was not possible to record due to the imidazolium ring yielding very intense signals and consequently is technically unfeasible. Therefore, the CD spectra of IFN-α 2b at 100 μg/mL in 150 mM NaH_2_PO_4_ (pH = 7.4) and of IFN-α 2b with 248 mM of [N_1112(OH)_][C_4_F_9_SO_3_] were measured in this work to infer the FIL influence on the protein secondary structure. The selected FIL concentration is above the ones that previously showed potential to unfold the protein in fluorescence spectroscopy studies. The CD spectra were recorded between 200 and 260 nm and are represented in Figure 8. The characteristic double minima around 208 and 222 nm are found in both CD spectra, and they almost overlap. The deconvolution of the spectra by the software K2D3 [47] yields a percentage of α helix content of 38.96 and 36.04 and β strand content of 14.71 and 13.63 for the IFN-α 2b and for IFN-α 2b + [N_1112(OH)_][C_4_F_9_SO_3_], respectively. The literature shows values of α helix content of around 50%; however, the variations in the experimental conditions, apparatus, and software used to deconvolute the secondary structure may justify these differences [65]. Thus, these results sustain the outcomes from the emission spectra, concluding that at this concentration, the interactions between the FIL and the biomolecule do not have a substantial effect on the secondary structure of IFN-α 2b. This behaviour is maintained for the concentrations of [N_1112(OH)_][C_4_F_9_SO_3_] equal to or lower than the one here tested.

#### 3.2.2. Binding Affinity

Microscale thermophoresis (MST) is an innovative approach that analyses the movement of fluorescent molecules out of microscopic temperature gradients in very reduced volumes, measuring precise binding events regardless of the size and physical properties of the target molecules. For that, it has been widely used in the detection of the binding of biomolecules, such as proteins, enzymes, and DNA to small molecules of interest such as ligands, substrates, and liposomes, among others. The binding of a protein to a ligand induces differences in its size, charge, and solvation energy, and these modifications are detected by thermophoresis. Even if that event produces reduced structural modifications, MST can detect the binding due to the induced alterations in the solvation entropy of the molecules, being a very sensitive method. The changes provoked by the binding in the thermophoresis of the fluorescent molecules can be then used to obtain the equilibrium dissociation constant, *K*_d_, by plotting the normalized fluorescence, *F*_norm_, of the labelled molecules *versus* the logarithm of the concentration of the ligand and fitting the binding curves with the models provided by the software [66,67]. This technique was previously applied in the determination of the binding affinity between ILs and lysozyme [68], as well as for inferring the interactions of other types of interferons [69,70] and other proteins [67,71,72] with different types of ligands.

In this work, a commercial kit was used to fluorescently tag the IFN-α 2b. In the process of labelling, commonly, one amine per protein is labelled, statistically distributing the position of the dye. Therefore, the risk of the label impairing the binding is very low, due to the typical number of lysine residues in proteins [67,71,72]. The [C_2_C_1_Im][C_4_F_9_SO_3_] and [N_1112(OH)_][C_4_F_9_SO_3_] were selected to study the binding with the IFN-α 2b. The solutions were prepared with a concentration of IFN-α 2b fixed at 2.7 μM in 5 mM of NaH_2_PO_4_ (pH = 7.4). As explained in the experimental section, in each run of MST, a maximum concentration is chosen and is consecutively diluted in 16 capillaries. Therefore, five different maximum concentrations of [C_2_C_1_Im][C_4_F_9_SO_3_] were selected to cover the range below the formation of the aggregates and to study the four distinct CACs. Only four maximum concentrations of [N_1112(OH)_][C_4_F_9_SO_3_] were chosen because this FIL only has three different CACs [38]. These experimental conditions do not allow the study of each phenomenon of aggregation individually above the 1st CAC once the range of dilutions of the solutions overlaps after this concentration. Thus, the determined dissociation constants *K*_d_ for each run that yields binding are influenced by the several stages of aggregation, which make it impossible to attribute the value to a specific CAC. Nevertheless, the *K*_d_ comprise the influence of the different aggregates of a FIL on the binding affinity with IFN-α 2b, and it will be only considered for qualitative analysis.

**Figure 9:**
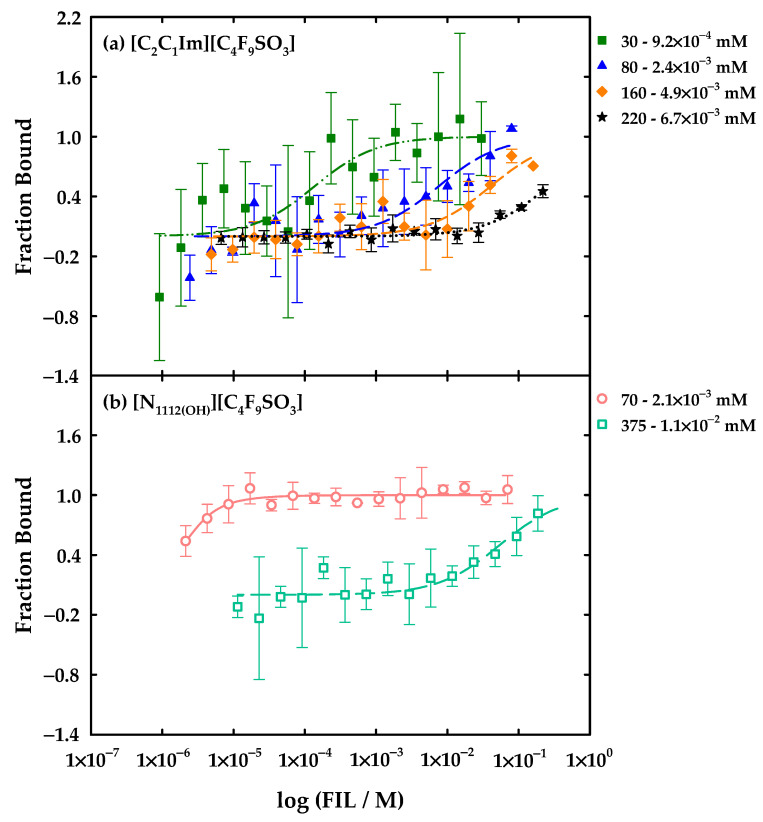
MST analysis of the interaction between the labelled IFN-α at 2.7 μM and the different concentrations of (**a**) [C_2_C_1_Im][C_4_F_9_SO_3_] and (**b**) [N_1112(OH)_][C_4_F_9_SO_3_] that yield binding affinity. All the samples were measured in 5 mM NaH_2_PO_4_ (pH = 7.4) at 25 °C. The error bars represent the standard deviations from the triplicate assays.

Figure S7 of Supplementary Materials shows the results for the nine assays. In the case of [C_2_C_1_Im][C_4_F_9_SO_3_] (Figure S7a of Supplementary Materials), it was possible to observe binding between the IFN-α 2b and the FIL in all the runs representing the several stages of aggregation. For the range of concentrations under the 1st CAC, only FIL monomers in aqueous solution, no binding was detected. The [N_1112(OH)_][C_4_F_9_SO_3_] results, illustrated in Figure S7b of Supplementary Materials, show that binding only occurs for the runs where concentrations were above the 2nd and 3rd CAC. Therefore, the *K*_d_ was determined in the cases where the binding was found, and the results are depicted in Table 2. The dose-response curves that were fitted for *K*_d_ determination are represented in Figure 9, plotting the fraction bound *versus* the concentration of the ligand. Analysing the results for the case of [C_2_C_1_Im][C_4_F_9_SO_3_], Figure 9a shows that the *K*_d_ increases as a result of the presence of different types of FILs aggregates in the following order: 1st CAC < 2nd CAC < 3rd CAC < 4th CAC, indicating a decrement in binding affinity between the protein and the FIL aggregates. Therefore, the type of aggregates formed at lower concentrations of FIL has a stronger affinity with this protein. The same behaviour is found in the case of [N_1112(OH)_][C_4_F_9_SO_3_], represented in Figure 9b, where an increased *K*_d_ is determined for the assay that comprises a higher concentration of FIL. This can also be verified by the deviation of the typical sigmoidal dose-response to the left, indicating a higher association of the ligand with the target molecule [71,72].

The comparison between the cations shows that [N_1112(OH)_]^+^ has more binding affinity with IFN-α 2b than the [C_2_C_1_Im]^+^, demonstrated by the lower *K*_d_ values and bigger left shift of the sigmoidal. Nevertheless, the [C_2_C_1_Im]^+^ showed a binding affinity with the protein in all stages of aggregation. However, the same behaviour was not verified for [N_1112(OH)_]^+^. Then, a higher range of concentrations, and subsequently different types of aggregates, can be used to design a proper system for IFN-α 2b delivery with the imidazolium-based FIL. Furthermore, this behaviour allows the usage of this FIL at lower concentrations, which is highly advantageous from a biomedical perspective. Crossing the results with the ones obtained in the emission analysis of the protein, it can be concluded that the interactions found under the 1st CAC are not as strong as the ones that occur above the 1st CAC. Moreover, the stronger affinity of the cholinium cation can explain the unfolding events found for higher concentrations of this FIL (Figure 7b). The interference of protein labelling should be ruled out in future studies, using fluorescence-labelling-free techniques, such as isothermal titration calorimetry, to support these results and enable a more specific study of the binding protein-FIL for each CAC. Nevertheless, this technique has provided valuable information on a strong binding between the aggregates of both studied FILs and IFN-α 2b, supporting the results previously discussed.

In conclusion, IFN-α 2b forms a complex (like a conjugate) with the FILs aggregates, that induces changes in the packaging, formation spontaneity, surface activity of the FILs aggregates, and size reduction of the protein aggregates, preventing its aggregation. The significant modifications in the morphology and size of FIL aggregates most likely occur due to the spatial adjustment resulting from the presence of IFN-α 2b. Moreover, strong interactions between the FIL aggregates and IFN-α 2b were disclosed using spectroscopy techniques and by determining their binding affinity, which is highly dependent on the different types of FIL aggregates and influenced by the nature of the cation.

To the best of our knowledge, there are no studies in the literature that use ILs as delivery systems for this protein. Nevertheless, Castro et al. have used several ILs to increase the efficiency of extraction of IFN-α 2b using several imidazolium-based ILs. The analysis of protein stability also yields prevention of the protein aggregation (no dimers were found) and the secondary structure of IFN-α 2b was not affected by the ILs [45]. Our results also indicate a prevention of the formation of protein aggregates. Therefore, the imidazolium cation seems to have a very positive effect on the stabilization of this specific protein. Looking to other types of compounds close to the IL family of compounds, one study has successfully used deep eutectic solvents based on natural sources for the thermal stabilization of this protein [73], which could be another interesting application for future work.

Finally, the use of FILs can be further tailored for the formulation of efficient delivery systems to transport and stabilize IFN-α 2b. This study constitutes an initial step in the direction of designing proper compounds to enable the encapsulation of IFN-α 2b, as previously obtained for other biomolecules.

## 4. Conclusions

In this study, a first step toward the use of FILs as drug delivery systems of the protein IFN-α 2b has been taken. The aggregation behaviour of the [C_2_C_1_Im][C_4_F_9_SO_3_] and [N_1112(OH)_][C_4_F_9_SO_3_] was studied to understand how the presence of the IFN-α 2b may alter the formation of aggregates and their properties, such as size and morphology. Then, the critical aggregation concentrations of [C_2_C_1_Im][C_4_F_9_SO_3_] were determined and it was concluded that the protein does not impair the formation of the three CACs characteristic of this FIL. The parameters of aggregation have shown that the presence of the protein can alter the packaging of the aggregates and the spontaneity of the process of aggregation, which can indicate an accommodation of the IFN-α 2b in the FILs aggregates. The surface properties of [C_2_C_1_Im][C_4_F_9_SO_3_] demonstrated that the IFN-α 2b highly influences the surface activity of this FIL, by the formation of more breaking points in the surface tension and contact angle profiles. Moreover, the critical packing parameters have shown that the conformation of the aggregates is altered in the presence of the protein, demonstrating a close interaction between FIL and protein. The further characterization of the morphology of the FILs aggregates proved that the aggregation of the protein is reduced, and the morphology of FIL aggregates is also modified, supporting that protein and FIL form a conjugate.

Finally, the protein-FIL interactions were studied in this paper by several spectroscopy techniques. The overall results allow us to conclude that a very strong interaction between the FILs aggregates of either [C_2_C_1_Im][C_4_F_9_SO_3_] and [N_1112(OH)_][C_4_F_9_SO_3_] and IFN-α 2b occurs, without altering the secondary structure of the protein. Only in the case of [N_1112(OH)_][C_4_F_9_SO_3_] was an alteration in the structure of the protein found, but in very high concentrations of FIL, which will not be used in biomedical applications. Furthermore, the determination of binding between protein and FIL has established and supported the complexation and formation of a conjugate that can contribute to the transport of the protein and its usage in drug delivery systems. More studies need to be pursued to fully understand the mechanism behind the formation of this complex.

This work represents a first and crucial step for the investigation of the discovery of the interactions between FILs and therapeutic proteins, as well as the formulation of feasible FILs-based DDSs, opening new avenues for the application of these biomaterials in the pharmaceutical field.

## Figures and Tables

**Table 1:** The chemical structure and acronyms of the fluorinated ionic liquids studied in this work.

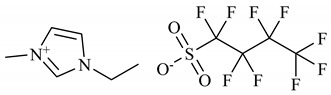 **[C_2_C_1_Im][C_4_F_9_SO_3_]** **1-Ethyl-3-methylimidazolium perfluorobutanesulfonate**	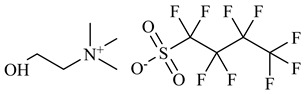 **[N_1112(OH)_][C_4_F_9_SO_3_]** **(2-Hydroxyethyl)trimethylammonium perfluorobutanesulfonate**

**Table 2:** Determined dissociation constants *K*_d_ by the analysis of the fitting of the MST dose-curve responses.

FIL	Assay	*K*_d_ (mM)
[C_2_C_1_Im][C_4_F_9_SO_3_]	30 − 9.2 × 10^−4^ mM	0.136 ± 0.117 ^a^
	80 − 2.4 × 10^−3^ mM	7.53 ± 5.61 ^a^
	160 − 4.9 × 10^−3^ mM	40.5 ± 29.5 ^a^
	220 − 6.7 × 10^−3^ mM	263 ± 197 ^a^
[N_1112(OH)_][C_4_F_9_SO_3_]	70 − 2.1 × 10^−3^ mM	5.97 × 10^−4^ ± 3.93 × 10^−4 a^
	375 − 1.1 × 10^−2^ mM	54.7 ± 36.9 ^a^

^a^ These values represent the *K*_d_ confidence of the fitting.

## Data Availability

Not applicable.

## Supplementary Materials

The following supporting information can be downloaded at: https://www.mdpi.com/article/10.3390/nano12111851/s1, Figure S1: Conductivity profile; Figures S2 and S3: Drops measured in goniometer; Figures S4 and S5: EDS analysis; Figure S6: Fluorescence spectra; Figure S7: Dose-response curve from MST analysis; Table S1: Critical aggregation concentrations, ionization degree, and Gibbs free energy of aggregation; Table S2: Density values; Table S3: Surface properties and critical packing parameters; Table S4: Polydispersity index.
