# Supplementary material for: Disclosing the Potential of Fluorinated Ionic Liquids as Interferon-Alpha 2b Delivery Systems
Source: Nanomaterials (Basel). 2022 May 28;12(11):1851. doi: 10.3390/nano12111851 (PMC9181987; doi:10.3390/nano12111851)
Supplement: Supplementary file 1 [file nanomaterials-12-01851-s001.zip › nanomaterials-1740861-supplementary.pdf]

nanomaterials

Supplementary Materials

Disclosing the Potential of Fluorinated Ionic Liquids as Interferon-Alpha 2b Delivery Systems

Margarida L. Ferreira, Nicole S. M. Vieira, Ana L. S. Oliveira, João M. M. Araújo * and Ana B. Pereiro *

LAQV, REQUIMTE, Department of Chemistry, NOVA School of Science and Technology, NOVA University Lisbon, 2829-516 Caparica, Portugal; mal.ferreira@campus.fct.unl.pt (M.L.F.); ns.vieira@campus.fct.unl.pt (N.S.M.V.); als.oliveira@campus.fct.unl.pt (A.L.S.O.)
* Correspondence: jmmda@fct.unl.pt (J.M.M.A.); anab@fct.unl.pt (A.B.P.); Tel.: +351-21-294-8300

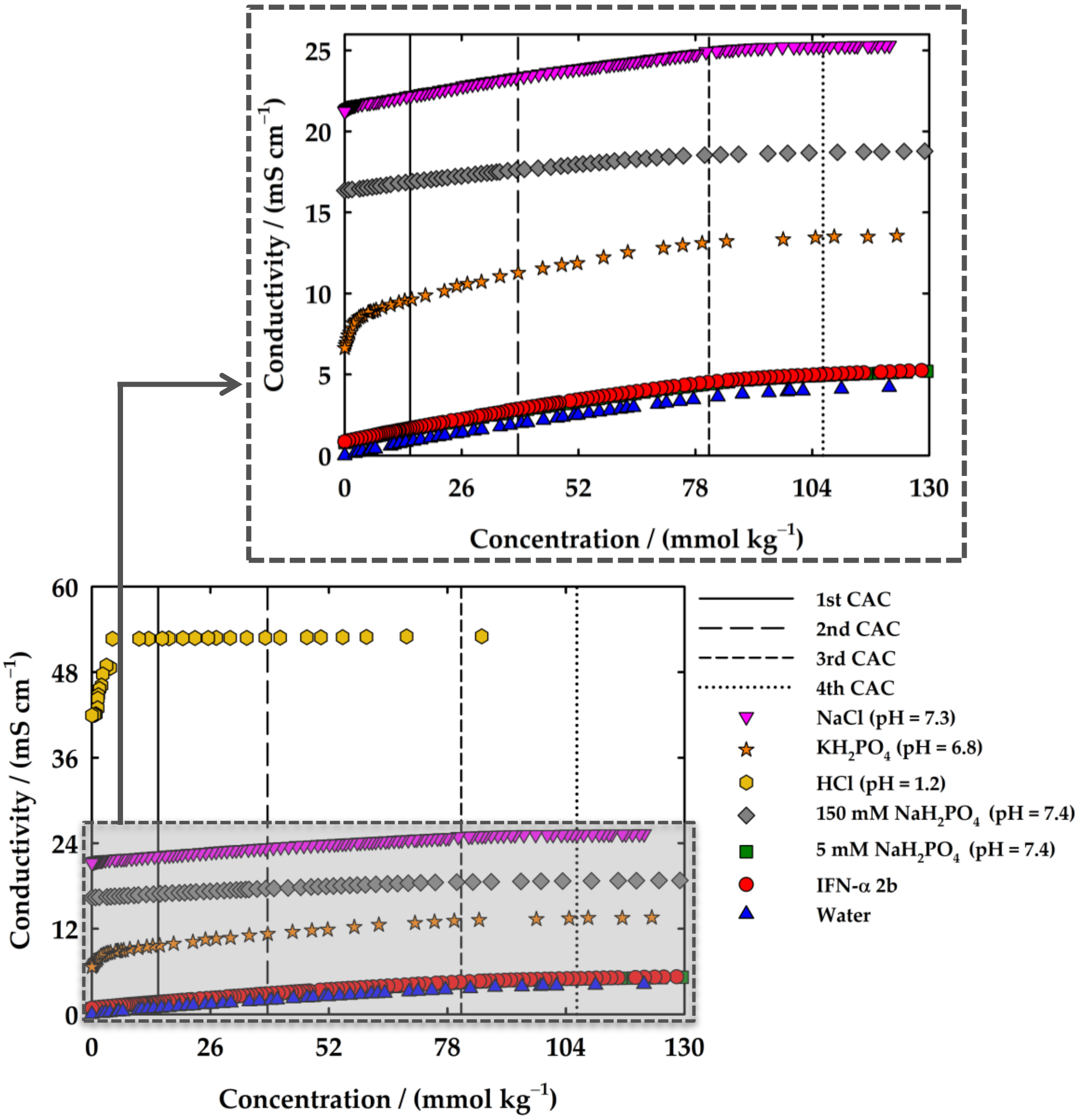

Figure S1. Conductivity profile of $[C_2C_1Im][C_4F_9SO_3]$ at 25°C from 0 to ~130 mM at different conditions: (i) 150 mM of NaCl at pH=7.3 (pink, ▼); (ii) 25 mM of KH_2PO_4 at pH=6.8 (orange, ★); (iii) 100 mM of HCl at pH 1.2 (yellow, ●); (iv) 150 mM of NaH_2PO_4 at pH 7.4 (grey, ◆); (v) 5 mM of NaH_2PO_4 at pH 7.4 (green, ■); (vi) 5 mM of NaH_2PO_4 at pH 7.4 with 10 µg/mL of IFN-α 2b (red, ●); and (vii) water (blue, ▲) [1] to comparison purposes. The lines represent the different CACs of $[C_2C_1Im][C_4F_9SO_3]$ in water [1].

Table S1. Critical aggregation concentrations, CACs, ionization degree, α, and Gibbs free energy of aggregation, ΔG^0_{agg}, of the systems with $[C_2C_1Im][C_4F_9SO_3]$ determined by conductometry at 25°C

		**150 mM NaCl (pH=7.3)**	**25 mM KH_2PO_4 (pH=6.8)**	**100 mM HCl (pH=1.2)**	**150 mM NaH_2PO_4 (pH=7.4)**	**5 mM NaH_2PO_4 (pH=7.4)**	**10 µg/mL IFN-α 2b + 5 mM NaH_2PO_4 (pH=7.4)**	**Water(a)**
**First CAC**	w_{FIL}	0.0014	0.0008	0.0019	0.0060	0.0056	0.0062	0.0060
	mmol kg^{-1}	3.51	1.99	4.65	14.57	13.75	15.19	14.55
	α	0.14	0.24	0.003	0.85	0.85	0.87	0.79
	ΔG^0_{agg} [kJ mol^{-1}]	-44.6	-44.6	-46.4	-23.4	-23.7	-23.0	-24.7
**Second CAC**	w_{FIL}	0.0138	0.0158		0.0135	0.0189	0.0179	0.0158
	mmol kg^{-1}	33.69	38.64		32.93	46.13	43.57	38.54
	α	0.75	0.66		0.82	0.85	0.85	0.84
	ΔG^0_{agg} [kJ mol^{-1}]	-22.9	-24.1		-21.6	-20.2	-20.3	-20.8
**Third CAC**	w_{FIL}	0.0342	0.0293		0.0282	0.0341	0.0342	0.0332
	mmol kg^{-1}	83.46	71.45		68.76	83.13	83.44	81.03
	α	0.17	0.18		0.21	0.34	0.52	0.29
	ΔG^0_{agg} [kJ mol^{-1}]	-29.3	-29.9		-29.5	-26.6	-23.8	-27.5

(a) Data from reference [1].

Table S2. Density, ρ, and mass fraction, w_{FIL}, for the binary system $[C_2C_1Im][C_4F_9SO_3]$ + water at 25°C.

w_{FIL}	ρ / g·cm^{-3}	**Reference**
1.0000	1.5458	[1]
0.9804	1.5257	[1]
0.9593	1.5060	[1]
0.9418	1.4949	[1]
0.9212	1.4765	[1]
0.8990	1.4615	[1]
0.8686	1.4421	[1]
0.8398	1.4270	[1]
0.7997	1.4049	[1]
0.7801	1.3914	[1]
0.7398	1.3603	[1]
0.6998	1.3417	[1]
0.6496	1.3081	[1]
0.5999	1.2773	[1]
0.5489	1.2462	[1]
0.5001	1.2177	[1]
0.2989	1.1135	This work
0.1000	1.0353	This work
0.0384	1.0104	This work
0.0245	1.0048	This work
0.0109	0.9994	This work
0.0030	0.9965	This work

Table S3. Critical aggregation concentration (CAC), surface properties, and critical packing parameters for [C_2C_1Im][$C_4F_9SO_3$] aqueous solutions determined by the tensiometer and goniometer at 25°C.

		5 mM NaH_2PO_4 (pH=7.4)	5 µg/mL IFN-α 2b + 5 mM NaH_2PO_4 (pH=7.4) Breakpoints				Water[(a)]
			1st	2nd	3rd	4th	
**Tensiometer**	CAC (w_{FIL})	0.0330	0.0008	0.0025	0.0089	0.0335	0.0375
	CAC (mmol·kg^{-1})	80.35	1.963	6.105	21.61	81.59	91.39
	γ_{CAC} [mN m^{-1}]	24.6	51.4	48.2	38.3	24.3	24.5
	$10^6\,\Gamma_{max}$ [mol m^{-2}]	3.26	–	–		4.41	2.60
	A_{min} [nm^2]	0.51	–	–		0.38	0.64
	Π_{CAC} [mN m^{-1}]	46.4	–	–		37.8	44.4
	ΔG^0_{ad} [kJ mol^{-1}]	-41.0	–	–		-32.36	-44.5
**Critical packing parameters**	$P_{eq\,8}$ [(b)]	0.550	–	–		0.7439	0.439
	$P_{eq\,9}$ [(b)]	0.553	–	–		0.7481	0.442
**Goniometer**	CAC (w_{FIL})	0.0329	0.0009	0.0030	0.0157	0.0430	–
	CAC (mmol·kg^{-1})	80.27	2.101	7.288	38.18	104.7	–

(a) Parameters from reference [1]; (b) the critical packing parameters were obtained by Equations 8 and 9 of the paper.

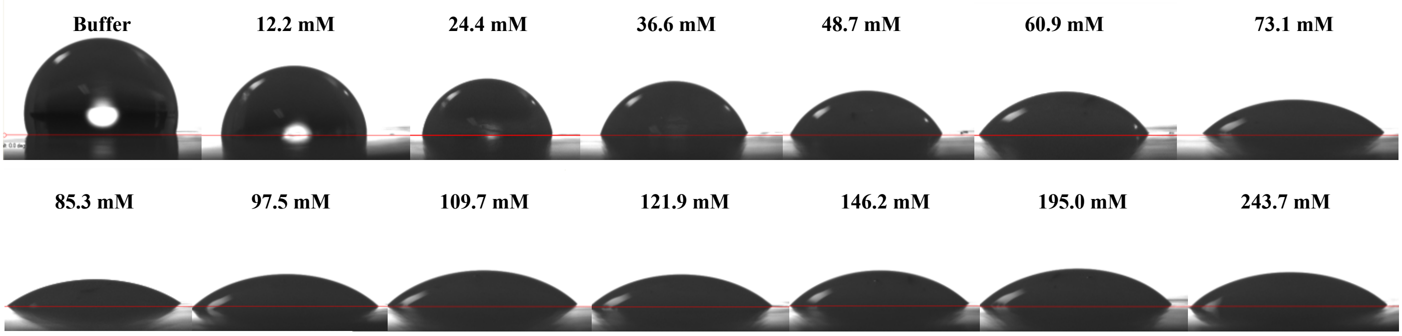

Figure S2. Drops measured in goniometer at 25°C of [C_2C_1Im][$C_4F_9SO_3$] aqueous solutions at different concentrations in 5 mM NaH_2PO_4 (pH = 7.4). The red line indicates the surface where the drop was deposit.

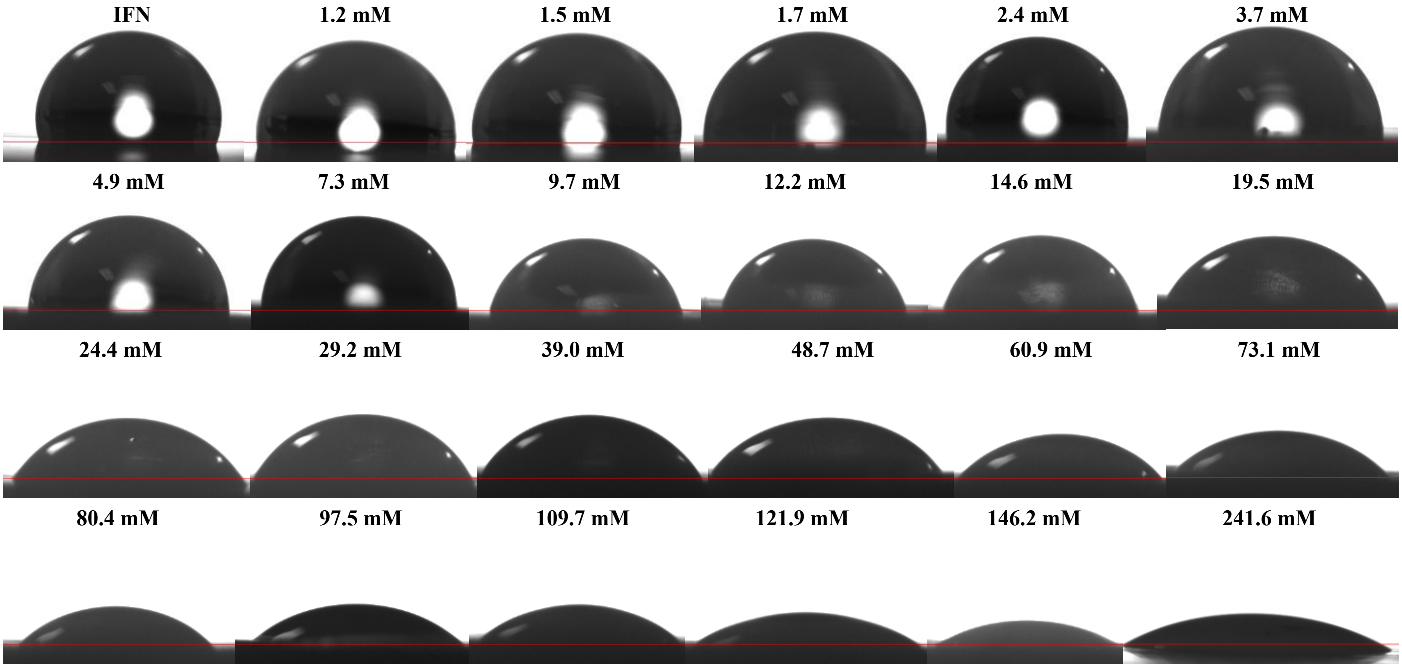

Figure S3. Drops measured in goniometer at 25°C of [C_2C_1Im][$C_4F_9SO_3$] aqueous solutions at different concentrations with 5 µg/mL of IFN-α 2b in 5 mM NaH_2PO_4 (pH=7.4). The red line indicates the surface where the drop was deposit.

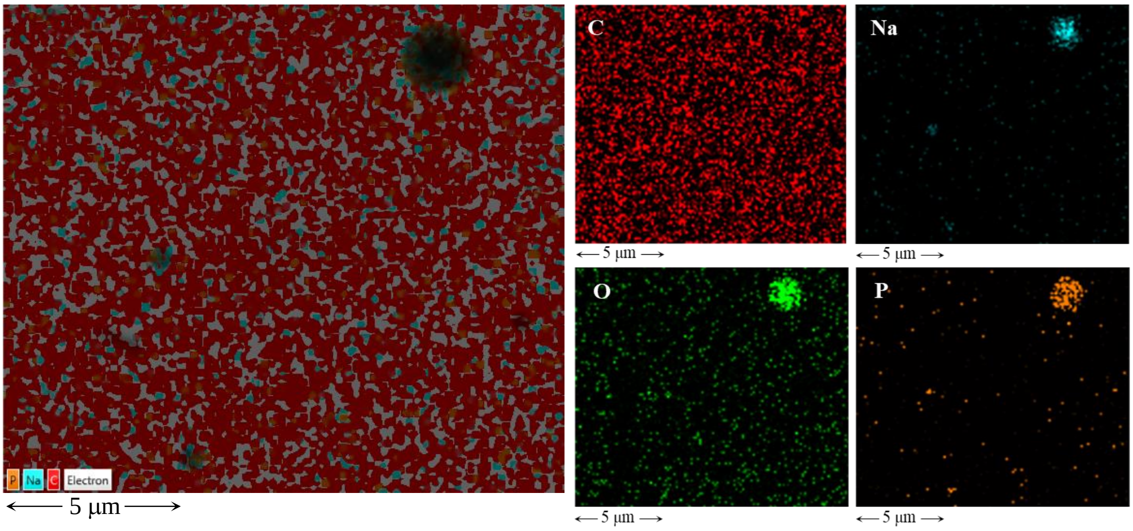

Figure S4. EDS analysis of IFN-α 2b at 0.0001 µg/mL in 150 mM NaH_2PO_4 measured at 25°C.

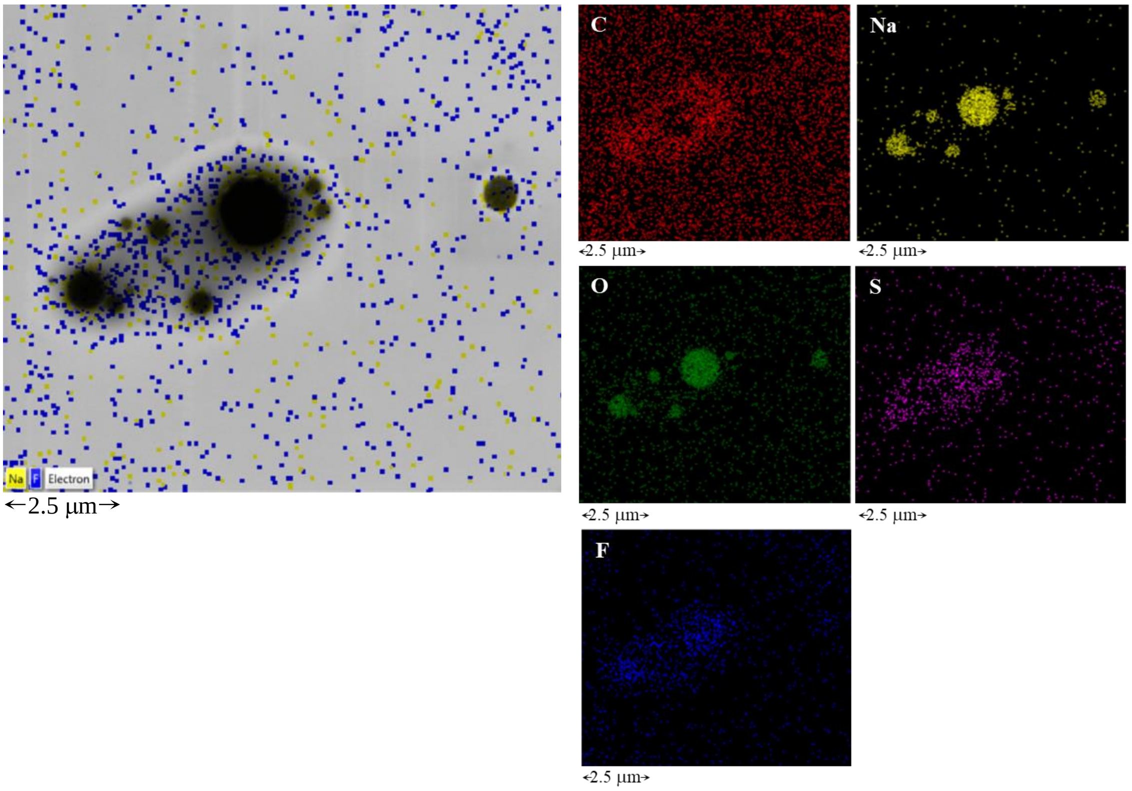

Figure S5. EDS analysis of $[C_2C_1Im][C_4F_9SO_3]$ at 29.2 mM in 150 mM NaH_2PO_4 measured at 25°C.

Table S4. Polydispersity index (PdI) obtained from DLS measurements in 150 mM NaH_2PO_4 measured at 25°C.

Sample	PDI
50 µg/mL IFNα-2b	0.427
29.3 mM $[C_2C_1Im][C_4F_9SO_3]$	1.000
29.3 mM $[C_2C_1Im][C_4F_9SO_3]$ + 50 µg/mL IFNα-2b	0.630
243.8 mM $[C_2C_1Im][C_4F_9SO_3]$	0.157
243.8 mM $[C_2C_1Im][C_4F_9SO_3]$ + 50 µg/mL IFNα-2b	0.279
29.8 mM $[N_{1112(OH)}][C_4F_9SO_3]$	0.784
29.8 mM $[N_{1112(OH)}][C_4F_9SO_3]$ + 50 µg/mL IFNα-2b	0.628
248.0 mM $[N_{1112(OH)}][C_4F_9SO_3]$	0.543
248.0 mM $[N_{1112(OH)}][C_4F_9SO_3]$ + 50 µg/mL IFNα-2b	0.849

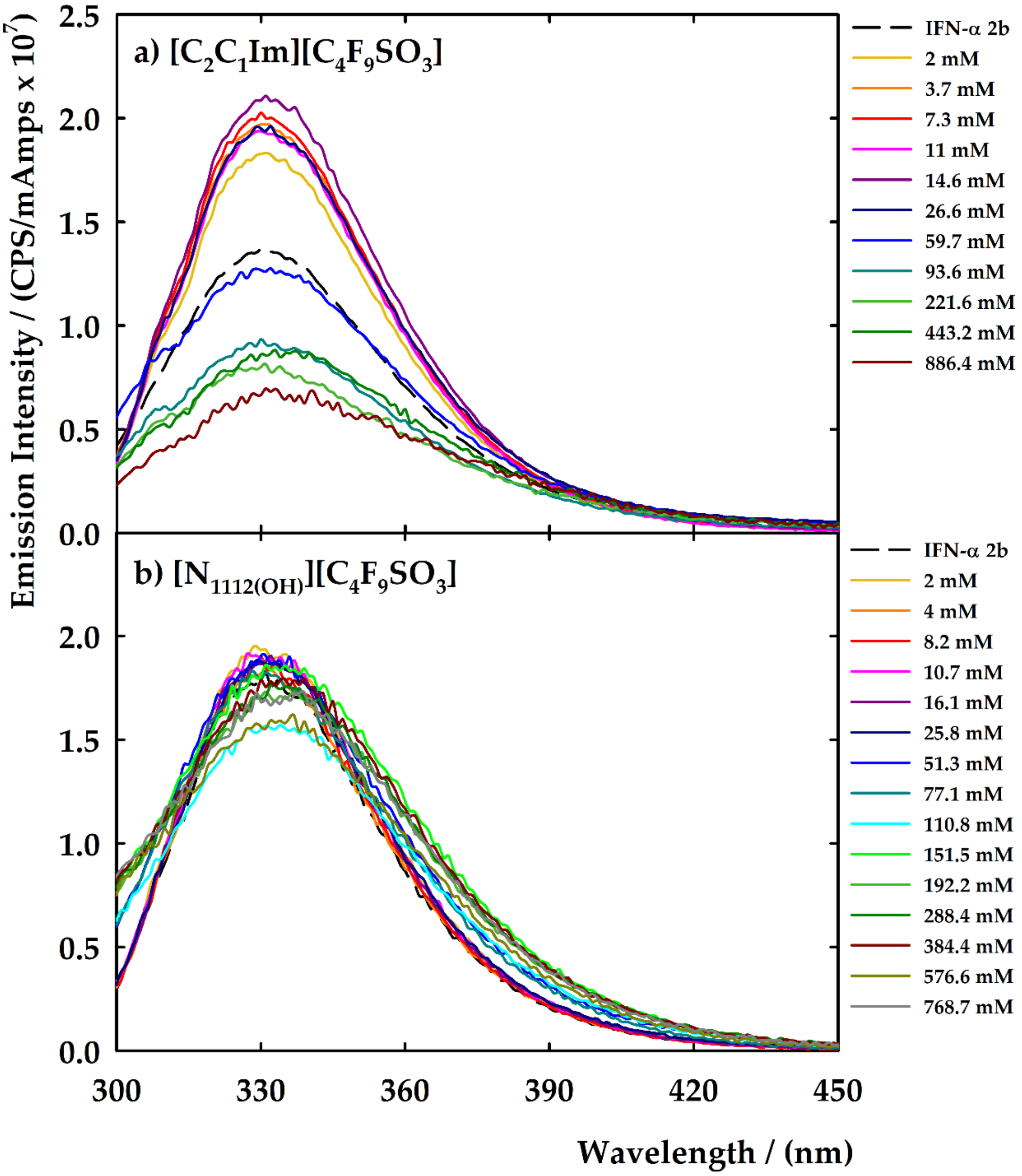

Figure S6. Fluorescence spectra of IFN-α 2b (20 µg/mL, black dashed line) with a) $[C_2C_1Im][C_4F_9SO_3]$ and b) $[N_{1112(OH)}][C_4F_9SO_3]$ at different concentrations. All samples were prepared in 5 mM NaH_2PO_4 at pH = 7.4 and measured at 25°C.

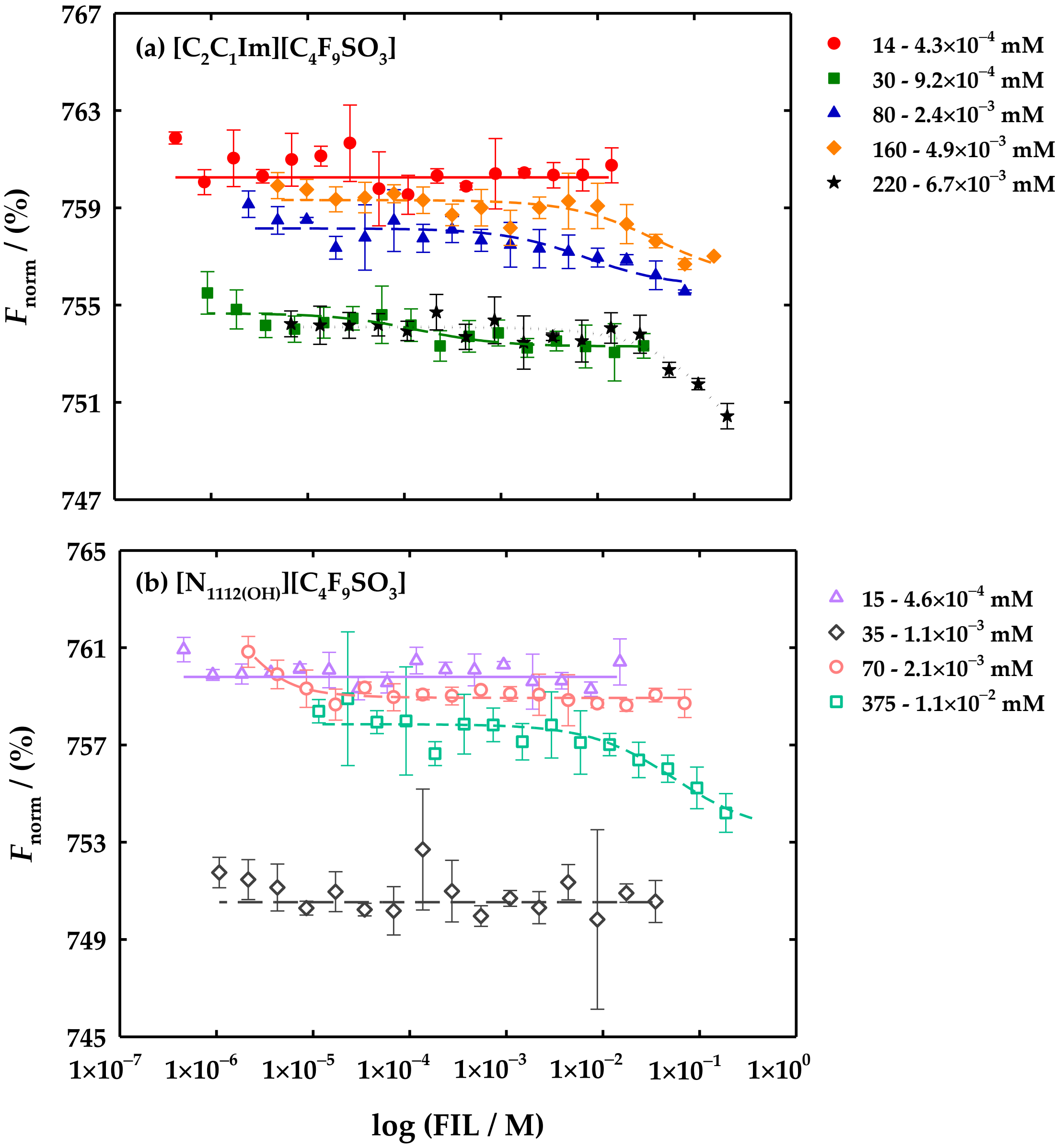

Figure S7. Dose-response curve from MST of labelled IFN-α at 2.7μM in the presence of different ranges of concentrations of a) $[C_2C_1Im][C_4F_9SO_3]$ and b) $[N_{1112(OH)}][C_4F_9SO_3]$. All the samples were measured in 5 mM NaH_2PO_4 (pH = 7.4) at 25°C. The error bars represent the standard deviations from the triplicate assays.

References

1. Pereiro, A.B.; Araújo, J.M.M.; Teixeira, F.S.; Marrucho, I.M.; Piñeiro, M.M.; Rebelo, L.P.N. Aggregation behavior and total miscibility of fluorinated ionic liquids in water. *Langmuir* **2015**, *31*, 1283–1295. https://doi.org/10.1021/la503961h (reference 38 of the paper)
